# Interferon regulatory factor-5-dependent CD11c^+^ macrophages contribute to the formation of rupture–prone atherosclerotic plaques

**DOI:** 10.1093/eurheartj/ehab920

**Published:** 2022-02-09

**Authors:** Andreas Edsfeldt, Maarten Swart, Pratibha Singh, Lea Dib, Jiangming Sun, Jennifer E Cole, Inhye Park, Dania Al-Sharify, Ana Persson, Mihaela Nitulescu, Patricia Das Neves Borges, Christina Kassiteridi, Michael E Goddard, Regent Lee, Petr Volkov, Marju Orho-Melander, Lars Maegdefessel, Jan Nilsson, Irina Udalova, Isabel Goncalves, Claudia Monaco

**Affiliations:** Department of Clinical Sciences, Clinical Research Center, Lund University, Malmö, Sweden; Department of Cardiology, Skåne University Hospital, Lund/Malmö, Sweden; Kennedy Institute of Rheumatology, Nuffield Department of Orthopaedics, Rheumatology and Musculoskeletal Sciences, University of Oxford, Roosevelt Drive, Headington, Oxford, UK; Wallenberg Center for Molecular Medicine, Lund University, Malmö, Sweden; Kennedy Institute of Rheumatology, Nuffield Department of Orthopaedics, Rheumatology and Musculoskeletal Sciences, University of Oxford, Roosevelt Drive, Headington, Oxford, UK; Department of Clinical Sciences, Clinical Research Center, Lund University, Malmö, Sweden; Kennedy Institute of Rheumatology, Nuffield Department of Orthopaedics, Rheumatology and Musculoskeletal Sciences, University of Oxford, Roosevelt Drive, Headington, Oxford, UK; Department of Clinical Sciences, Clinical Research Center, Lund University, Malmö, Sweden; Kennedy Institute of Rheumatology, Nuffield Department of Orthopaedics, Rheumatology and Musculoskeletal Sciences, University of Oxford, Roosevelt Drive, Headington, Oxford, UK; Kennedy Institute of Rheumatology, Nuffield Department of Orthopaedics, Rheumatology and Musculoskeletal Sciences, University of Oxford, Roosevelt Drive, Headington, Oxford, UK; Department of Clinical Sciences, Clinical Research Center, Lund University, Malmö, Sweden; Department of Clinical Sciences, Clinical Research Center, Lund University, Malmö, Sweden; Department of Clinical Sciences, Clinical Research Center, Lund University, Malmö, Sweden; Kennedy Institute of Rheumatology, Nuffield Department of Orthopaedics, Rheumatology and Musculoskeletal Sciences, University of Oxford, Roosevelt Drive, Headington, Oxford, UK; Kennedy Institute of Rheumatology, Nuffield Department of Orthopaedics, Rheumatology and Musculoskeletal Sciences, University of Oxford, Roosevelt Drive, Headington, Oxford, UK; Kennedy Institute of Rheumatology, Nuffield Department of Orthopaedics, Rheumatology and Musculoskeletal Sciences, University of Oxford, Roosevelt Drive, Headington, Oxford, UK; Nuffield Department of Surgical Sciences, Oxford, University of Oxford; Department of Clinical Sciences, Clinical Research Center, Lund University, Malmö, Sweden; Department of Clinical Sciences, Clinical Research Center, Lund University, Malmö, Sweden; Department of Medicine, Karolinska Institute, Stockholm, Sweden; Department of Vascular and Endovascular Surgery, Technical University Munich and DZHK Partner Site Munich, Munich, Germany; Department of Clinical Sciences, Clinical Research Center, Lund University, Malmö, Sweden; Kennedy Institute of Rheumatology, Nuffield Department of Orthopaedics, Rheumatology and Musculoskeletal Sciences, University of Oxford, Roosevelt Drive, Headington, Oxford, UK; Department of Clinical Sciences, Clinical Research Center, Lund University, Malmö, Sweden; Department of Cardiology, Skåne University Hospital, Lund/Malmö, Sweden; Kennedy Institute of Rheumatology, Nuffield Department of Orthopaedics, Rheumatology and Musculoskeletal Sciences, University of Oxford, Roosevelt Drive, Headington, Oxford, UK

**Keywords:** IRF5, Macrophages, Atherosclerosis, Plaque rupture

## Abstract

**Aims:**

Inflammation is a key factor in atherosclerosis. The transcription factor interferon regulatory factor-5 (IRF5) drives macrophages towards a pro-inflammatory state. We investigated the role of IRF5 in human atherosclerosis and plaque stability.

**Methods and results:**

Bulk RNA sequencing from the Carotid Plaque Imaging Project biobank were used to mine associations between major macrophage associated genes and transcription factors and human symptomatic carotid disease. Immunohistochemistry, proximity extension assays, and Helios cytometry by time of flight (CyTOF) were used for validation. The effect of IRF5 deficiency on carotid plaque phenotype and rupture in ApoE^−/−^ mice was studied in an inducible model of plaque rupture. Interferon regulatory factor-5 and ITGAX/CD11c were identified as the macrophage associated genes with the strongest associations with symptomatic carotid disease. Expression of IRF5 and ITGAX/CD11c correlated with the vulnerability index, pro-inflammatory plaque cytokine levels, necrotic core area, and with each other. Macrophages were the predominant CD11c-expressing immune cells in the plaque by CyTOF and immunohistochemistry. Interferon regulatory factor-5 immunopositive areas were predominantly found within CD11c^+^ areas with a predilection for the shoulder region, the area of the human plaque most prone to rupture. Accordingly, an inducible plaque rupture model of ApoE^−/−^Irf5^−/−^ mice had significantly lower frequencies of carotid plaque ruptures, smaller necrotic cores, and less CD11c^+^ macrophages than their IRF5-competent counterparts.

**Conclusion:**

Using complementary evidence from data from human carotid endarterectomies and a murine model of inducible rupture of carotid artery plaque in IRF5-deficient mice, we demonstrate a mechanistic link between the pro-inflammatory transcription factor IRF5, macrophage phenotype, plaque inflammation, and its vulnerability to rupture.

## Structured Graphical Abstract

**Figure:**
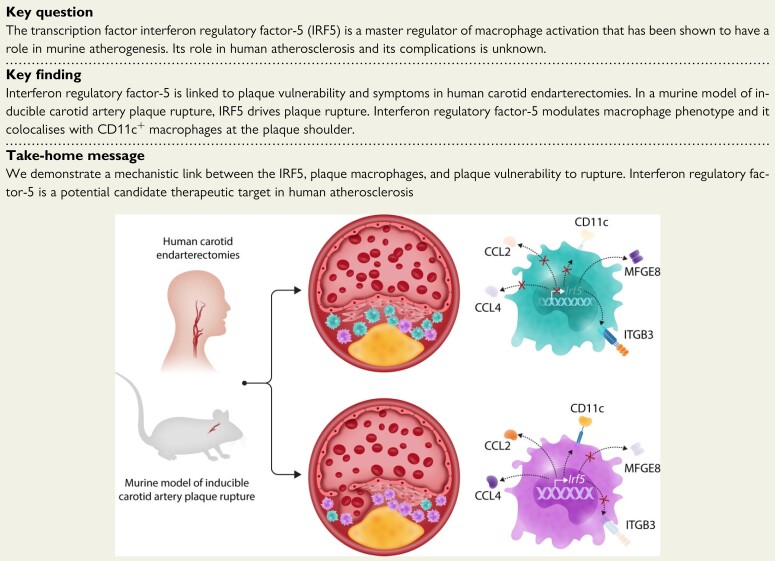
Macrophages perform both pro- and anti-atherogenic functions depending on their transcriptional status and activation. Using human carotid endarterectomies and a murine model of inducible rupture of carotid plaque, we show that the transcription factor interferon regulatory factor-5 (IRF5) promotes macrophage activation with the production of chemokines CCL2 and CCL4, and the expression of the integrin CD11c ,while it disables the production of proteins required for the uptake of apoptotic cells within the plaque (efferocytosis) such as MFEG8 and the integrin ITGB3. In doing so, IRF5 promotes inflammation and increases the necrotic core size enhancing plaque vulnerability to rupture. MFGE8, Milk fat globule—epidermal growth factor—factor VIII; CCL2, C–C motif chemokine ligand 2; CCL4, C–C motif chemokine ligand 4; ITGB3, Integrin subunit beta 3.

**See the editorial comment for this article ‘Advancing therapeutic targeting of the vulnerable plaque’, by Alexandra A.C. Newman *et al*., https://doi.org/10.1093/eurheartj/ehac060.**

**Translational perspective**

Macrophages perform both pro- and anti-atherogenic functions depending on their programming. Rewiring macrophage transcriptional states is an attractive therapeutic strategy for cardiovascular disease. The transcription factor interferon regulatory factor-5 (IRF5) is a master regulator of macrophage activation, important in murine atherogenesis. Its role in human atherosclerosis and its complications are unknown. Using human carotid endarterectomies and a murine model of inducible rupture of carotid plaque, we here demonstrate that IRF5 has a role in plaque vulnerability and rupture. Our study indicates that IRF5 is a candidate therapeutic target to lower the risk of plaque rupture by reducing macrophage activation and plaque inflammation.

## Introduction

Ischaemic stroke is one of the dominant causes of severe morbidity and premature death. Rupture of an atherosclerotic plaque is considered to be one of its major underlying causes.^1^ Lipoprotein retention and inflammation in the arterial wall both contribute to atherosclerotic plaque formation.^2^ The formation of rupture–prone plaques, so-called vulnerable plaques, is the result of cell death, impaired efferocytosis, and degradation of the stabilizing fibrous cap.^3^ Activation of the immune system not only contributes to atherogenesis but also to vulnerable plaque biology.^4^ Macrophages are key players in cardiovascular disease (CVD) and their polarization states have a role in atherogenesis.^5^ Different stimuli and transcription factors have been shown to regulate macrophage states *in vitro* and in murine models of atherogenesis.^5,6^ Yet, the transcription factors that drive macrophage activation in the human plaque and its rupture remain to be identified.

Interferon regulatory factor 5 (IRF5) is a master regulator of pro-inflammatory macrophages in mouse and human.^7–9^ We previously showed, in ApoE^−/−^ mice on a chow diet, that IRF5 drives atherogenesis and the formation of the necrotic core by impairing macrophage efferocytosis capacity.^10^ Conditional myeloid deficiency of IRF5 phenocopied the effect on murine atherosclerotic lesion stability and macrophage polarization, providing further insight on the importance of IRF5 in lesional macrophage proliferation and foam cell formation in ApoE^−/−^ mice with a high-cholesterol diet.^11^ Evidence for a role of IRF5 in human atherosclerosis and plaque rupture is so far lacking. Here, we show that IRF5 and CD11c expression is linked to symptomatic and vulnerable carotid plaques in humans, and that IRF5 drives pro-inflammatory CD11c^+^ macrophages and inducible plaque rupture in ApoE^−/−^ mice, demonstrating IRF5 as a candidate therapeutic target in human atherosclerosis.

## Methods

A fully detailed description can be found as Supplementary material online.

### Carotid plaque cohort

Human atherosclerotic carotid plaques were obtained from the Carotid Plaque Imaging Project (CPIP) biobank to study IRF5 and CD11c expression. All patients included in the CPIP cohort gave their informed consent to participate in the study. The study was approved by the ethical review board at Lund University and follows the declaration of Helsinki.

Plaques used included in the study were either associated with symptoms (stroke, transient ischaemic attack, or amaurosis fugax) with a degree of stenosis >70% (assessed by duplex ultrasound) or asymptomatic with a degree of stenosis >80%. All patients included were examined by a neurologist prior to surgery.

Clinical characteristics of the cohort, including information regarding smoking, hypertension (systolic blood pressure >140 mmHg), and the use of statins were recorded at inclusion. Plasma samples to assess glycated haemoglobin (HbA1c), creatinine, high-sensitivity C-reactive protein (hsCRP), and circulating lipoproteins were collected the day before surgery.

All collected plaque specimens were immediately snap frozen after surgical removal. A 1 mm fragment from the most stenotic region of the plaque was kept for histological analysis and the rest of the plaque was homogenized.^12^

### Human plaque RNA sequencing

Expression values of macrophage associated genes and transcription factors were evaluated from the global transcriptome RNA sequencing data of 60 carotid plaque samples.

### Human carotid plaque histology and immunofluorescence

The 1 mm segment from the most stenotic part of the plaque was cryo-sectioned into 8 µm sections. Sections were then stained for neutral lipids (Oil Red O), macrophages (CD68), smooth muscle cells (α-actin), intra-plaque haemorrhage (glycophorin A/CD235), and collagen (Russell-Movat pentachrome).

Vulnerability index was calculated as a ratio between the sum of lipids (Oil red O, % plaque area), macrophages (CD68, % plaque area), and intra-plaque haemorrhage (glycophorin A, % plaque area) divided by the sum of smooth muscle cells (α-actin, % plaque area) and collagen (Movat pentachrome, % plaque area).

Immunofluorescence staining was performed on 8 µm carotid sections embedded in optimal cutting temperature (OCT) compound using a cocktail of anti-CD11c (Abcam, ab11029) and anti-CD68 (cell signalling technology, clone D4B9C, #76437S) antibodies.

### Mass cytometry

Carotid endarterectomies from patients undergoing surgery for carotid artery disease for mass cytometry were obtained at John Radcliff Hospital, Oxford, UK. The protocol was approved by the Research Ethics Committee. Tissues were enzymatically digested and single cell suspension was generated as previously described.^13^

All directly conjugated antibodies were purchased from Fluidigm or conjugated in-house using MaxPar X8 Polymer Kits (Fluidigm) according to the manufacturer’s instructions (listed in Supplementary material online, *Table S1*). Samples were stained with rhodium DNA intercalator (Fluidigm) as a viability dye and Fc receptors were blocked (BD Biosciences) before cells were stained with the antibody mixture for 30 min at 4°C. The cells were then washed and incubated with Iridium DNA intercalator (Fluidigm) in Maxpar fix and perm buffer (Fluidigm) overnight at 4°C. Prior to acquisition, cells were washed with water (Fluidigm) and acquired on a Helios mass cytometer (Fluidigm). Data in the fcs files format were uploaded to Cytobank (www.cytobank.org) for gating and analysis using the automated dimensionality reduction algorithm viSNE. The gating strategy consisted of sequential gating for intact single cells using the iridium DNA intercalator, removal of the normalization beads, and gating for cell viability using the rhodium DNA intercalator. CD45^+^ cells were gated based on expression of CD45.

### Mouse model

*ApoE* ^−/−^ *Irf5* ^−/−^ mice were generated by breeding *ApoE* ^−/−^ mice (C57BL/6 background from Charles River Laboratories) in-house with IRF5^−/−^ mice on a C57BL/6 background. All mice were studied according to institutional guidelines and to UK Home Office regulations. Only male mice were used and all mice were negative for the Dock2 mutation. Mice were fed chow diet and were housed under specific pathogen-free conditions. A surgical model combining carotid artery ligation and placement of a shear stress modifying cast was used to study effect of IRF depletion in inducible plaque ruptures (Supplementary material online, *Figure S1*).

### Statistical analysis

CD11c and IRF5 plaque areas were non-normally distributed whereas *ITGAX* and *IRF5* gene expression were normally distributed according to Shapiro–Wilk test for normality. Variables are presented as median and interquartile range (IQR) or mean and standard deviation (SD) depending on distribution. Mann–Whitney *U* test (continuous data), Student’s *t*-test (continuous data), and *χ* ^2^ test (categorical data) were used for two group comparisons. Spearman’s rho and Pearson’s correlation coefficient were used for correlation analyses.

SPSS 22.0 (IBM Corp., Amonk, NY, USA) and GraphPad Prism 7 were used for statistical analysis. Probability values of *P* < 0.05 were considered statistically significant.

### Data availability

The data underlying this article will be shared on reasonable request and in compliance with the appropriate general data protection regulation (GDPR) regulations.

## Results

### Interferon regulatory factor 5 and CD11c gene expression are associated with symptomatic human carotid plaques

RNA sequencing was performed on RNA isolated on the most stenotic region of human atherosclerotic carotid plaques from the CPIP cohort. Patient clinical characteristics are presented in *Table 1*.

**Table 1:** Clinical characteristics of the 60 patients which plaques were used for RNA sequencing

	Asymptomatic (*n* = 26)	Symptomatic (*n* = 34)	*P*-value
Male sex	62%	59%	0.83
Age, years, median (IQR)	68 (60–70)	75 (68–80)	<0.001
Diabetes	19%	32%	0.26
BMI, kg/m^2^, median (IQR)	27 (24–30)	27 (24–29)	0.36
Current smoker	58%	23%	0.03
Hypertension	77%	68%	0.43
Medication			
Anti-hypertensive	81%	79%	0.9
Statins	88%	76%	0.23
Laboratory parameters, median (IQR)			
Total cholesterol (mmol/L)	4.3 (3.5–5.3)	4.5 (3.6–5.6)	0.67
LDL (mmol/L)	2.4 (2.0–3.1)	2.7 (2.1–3.9)	0.35
HDL (mmol/L)	1.1 (0.9–1.4)	1.1 (0.8–1.3)	0.36
Triglycerides (mmol/L)	1.3 (1.0–1.9)	1.6 (0.8–2.3)	0.89
Creatinine (mmol/L)	77 (70–88)	92 (71–113)	0.09
hsCRP (mg/L)	2.9 (1.5–6.4)	4.4 (2.1–9.6)	0.27

Mann–Whitney test or *χ* ^2^ test for categorical data.

IQR, interquartile range; BMI, body mass index; LDL, low-density lipoproteins; HDL, high-density lipoproteins; hsCRP, high-sensitivity C-reactive protein.

**Figure 1:**
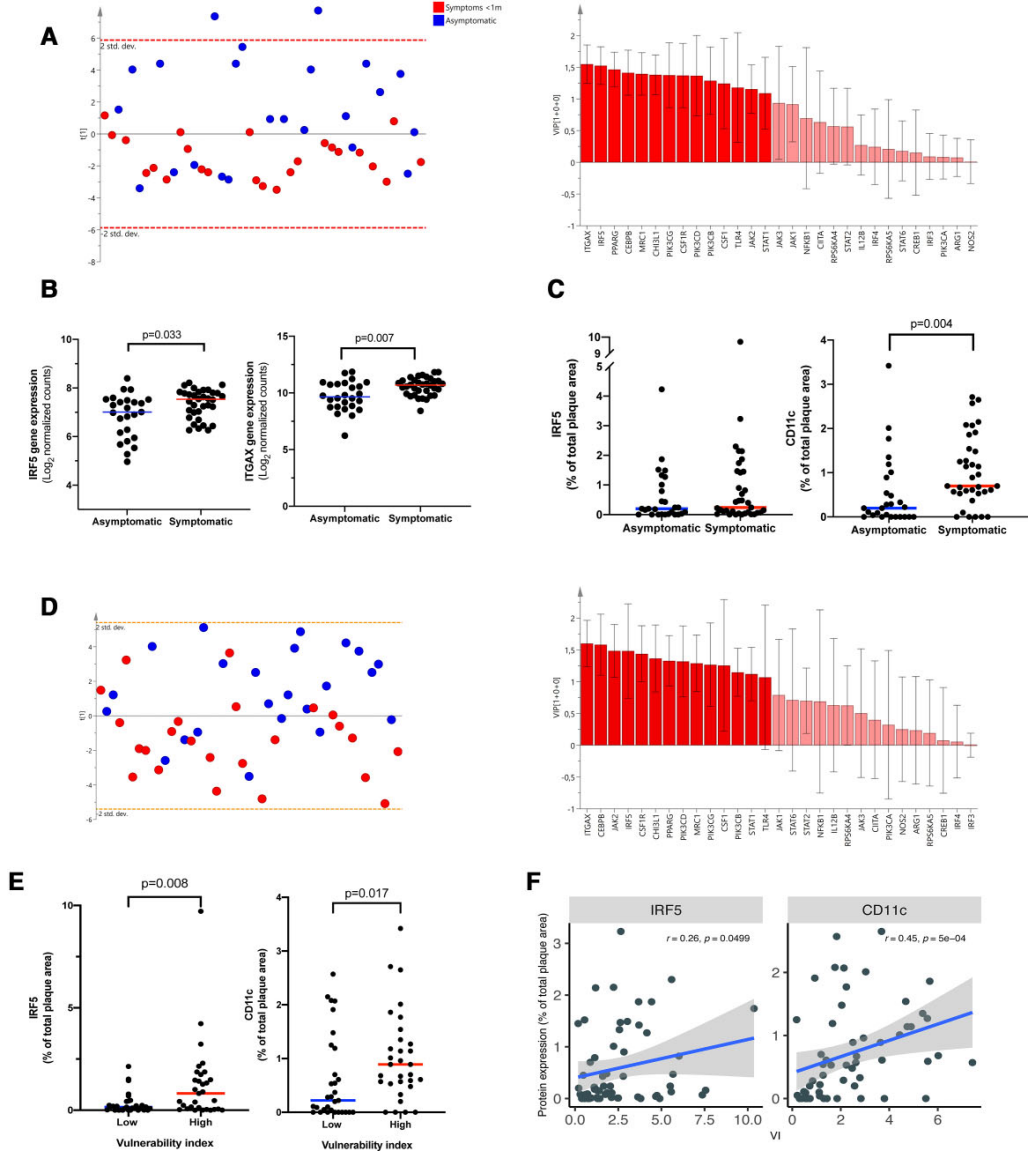
ITGAX (CD11c) gene expression and CD11c plaque area are associated with a vulnerable plaque phenotype and symptomatic carotid plaques in humans. (*A*) Using an OPLS-DA analysis, *IRF5* and *ITGAX* (CD11c) were identified as the myeloid cell surface marker and transcription factor and with the largest impact on separating symptomatic (<31 days prior to surgery) plaques from asymptomatic plaques. RNAseq data from 47 human carotid plaques (24 symptomatic and 23 asymptomatic). Blue indicates asymptomatic and red indicates symptomatic. (*B*) Human plaque gene expression levels of *IRF5* and *ITGAX* (CD11c) were also significantly increased in plaques associated with symptoms within 31 days prior to surgery compared with asymptomatic plaques. Lines indicate mean levels, each dot represents an individual value (*n* = 60, 34 symptomatic and 26 asymptomatic). (*C*) CD11c^+^ but not IRF5^+^ plaque area, as assessed by immunohistochemistry, was increased in symptomatic carotid plaques. Lines indicate median levels, each dot represents an individual value (*n* = 62, 35 symptomatic <31 days prior to surgery and 27 asymptomatic). Data are presented as % of total plaque area. (*D*) OPLS-DA analysis identified *ITGAX* as the myeloid cell gene with the largest impact on separating plaques with a vulnerability index above median from plaques with a vulnerability index below median (*n* = 47, 24 symptomatic and 23 asymptomatic). Blue indicates less than median vulnerability index and red indicates greater than median vulnerability index. (*E*) IRF5 and CD11c plaque areas are increased in plaques with a high (above median, *n* = 31) vulnerability index compared with plaques with a low vulnerability index (below median, *n* = 31). Mann–Whitney *U* tests were used. (*F*) Plaque area stained positive for CD11c and IRF5 correlated with the calculated vulnerability index (*n* = 62). Spearman test was used for the correlation analysis. CD11c, cluster of differentiation 11c; IRF5, interferon regulatory factor 5.

Amongst 29 commonly described macrophage associated markers and transcription factors, we set to identify the ones that could separate symptomatic (symptoms <31 days prior to surgery, *n* = 24) from asymptomatic plaques (*n* = 23) using the orthogonal projections to latent structures discriminant analysis (OPLS-DA) method.^6^ The OPLS-DA analysis revealed that symptomatic plaques were statistically distinguishable from asymptomatic based on selected markers (R^2^Y = 0.34, Q^2^Y = 0.2; CV ANOVA *P* = 0.008). *CD11c* (*ITGAX*) and *IRF5* were identified to be the strongest contributors to the separation (ranked highest on VIP score, *Figure 1A*). Gene expression levels of *IRF5* and *ITGAX* were also significantly higher in symptomatic compared with asymptomatic plaques [7.3 (SD 0.6) vs. 6.8 (SD 0.9) log_2_ normalized counts, *P* = 0.033 and 10.6 (SD 0.8) vs. 9.7 (SD 1.3) log_2_ normalized counts, *P* = 0.007; *n* = 60; *Figure 1B*]. No associations between IRF5 or CD11c protein expression and gender, age, body mass index, HbA1c, diabetes, total cholesterol, low-density lipoproteins, high-density lipoproteins, triglycerides, hsCRP, creatinine, or medications (lipid-lowering or anti-hypertensive) were identified.

### Interferon regulatory factor 5 and ITGAX were co-expressed in a gene cluster that is associated with symptomatic status

Based on the RNA-seq data, network clustering was performed. Six gene modules were identified (Supplementary material online, *Figure S2A*). *IRF5* and *ITGAX* were found in the blue module which composes of 482 genes in total. Interestingly, the first principal component of the gene expression of blue module showed positive correlation with symptomatic status (*r* = 0.39, *P* = 0.002, Supplementary material online, *Figure S2B*). Within the module, *ITGAX* and *IRF5* showed high intra-modular connectivity and relatively high gene significance in the association with symptomatic status and (Supplementary material online, *Figure S2C*). This gene cluster enriched in ‘proteins involved in non-alcoholic fatty liver disease’ (*P* = 6.9 × 10^−11^) and ‘proteins involved in atherosclerosis’ (*P* = 1.7 × 10^−9^) whereas IRF5 was absent in the enriched term. The top enriched pathway was ‘dendritic cells function in atherosclerosis’ (*P* = 0.002) when IRF5 was present.

### Interferon regulatory factor 5 and CD11c are associated with a vulnerable plaque phenotype in humans

Next, tissue sections from the most stenotic region of 62 carotid plaques (27 asymptomatic and 35 with symptoms <31 days prior to surgery, clinical characteristics are presented in *Table 2*) were stained for CD11c and IRF5. Plaque area stained positive for CD11c was significantly higher in the symptomatic than the asymptomatic plaques [0.7 (IQR 0.53–1.54)% vs. 0.2 (IQR 0–0.9)% of total plaque area, *P* = 0.004, *Figure 1C*] whereas no significant difference was identified in the area positive for IRF5 [0.24 (IQR 0.06–1.45)% vs. 0.2 (0.01–1.0)% of total plaque area, *P* = 0.32].

**Table 2:** Clinical characteristics of the 62 patients which plaques were used for immunohistochemical analyses of IRF5 and CD11c

	Asymptomatic (*n* = 27)	Symptomatic (*n* = 35)	*P*-value
Male sex	67%	63%	0.8
Age, years, median (IQR)	68 (63–70)	71 (64–78)	0.04
Diabetes	26%	40%	0.25
BMI, kg/m^2^, mean (IQR)	27 (24–29)	27 (24–30)	0.57
Current smoker	52%	23%	0.06
Hypertension	78%	77%	0.9
Medication			
Anti-hypertensive	81%	89%	0.4
Statins	93%	86%	0.4
Laboratory parameters, median (IQR)			
Total cholesterol (mmol/L)	4.1 (3.4–5.3)	4.2 (3.5–5.6)	0.8
LDL (mmol/L)	2.3 (1.9–3.2)	2.7 (2.0–3.9)	0.4
HDL (mmol/L)	0.9 (0.8–1.4)	1.1 (0.8–1.2)	0.9
Triglycerides (mmol/L)	1.3 (0.9–2.2)	1.7 (1.1–2.3)	0.3
Creatinine (mmol/L)	85 (74–94)	91 (70–107)	0.65
hsCRP (mg/L)	4.1 (1.8–6.8)	4.5 (1.8–6.7)	0.9

Mann–Whitney test or *χ* ^2^ test for categorical data.

IQR, interquartile range; BMI, body mass index; LDL, low-density lipoproteins; HDL, high-density lipoproteins; hsCRP, high-sensitivity C-reactive protein. Symptomatic means patients suffering from a transient ischaemic attack, stroke of amaurosis fugax <31 days prior to surgery.

We next sought to explore if IRF5 and CD11c were associated with the rupture–prone or vulnerable plaque phenotype.^3^ The human carotid plaques were classified into high or low vulnerability index based on their histological characteristics assessed on adjacent sections as previously described.^14^ In short, the sum of plaque area stained positive for macrophages (CD68), intra-plaque haemorrhage (glycophorin A), and lipids (Oil red O) was divided by the sum of plaque area stained positive for collagen (Movat pentachrome) and vascular smooth muscle cells (α-actin; Supplementary material online, *Figure S3*). Using an OPLS-DA analysis on the gene expression data, we identified the transcription factors and macrophage markers that had the greatest influence on separating plaques with a high vulnerability index from plaques with a low vulnerability index (above or below median; R^2^Y = 0.27, Q^2^Y = 0.18; *P* = 0.018). Importantly, IRF5 and CD11c were again identified to be amongst the strongest contributors for separating plaques with a more vulnerable plaque phenotype from stable plaques (ranked amongst top VIP scorers; *Figure 1D*). In line with this, CD11c and IRF5 immunopositive plaque areas were greater in plaques with a vulnerability index above median [0.8 (IQR 0.06–1.7) vs. 0.15 (IQR 0.01–0.43), *P* = 0.008 and 0.9 (IQR 0.5–1.4) vs. 0.2 (IQR 0.0–1.2), *P* = 0.017; *Figure 1E*, *n* = 47]. Interferon regulatory factor-5 and CD11c immunopositive plaque areas also correlated positively with the vulnerability index (*r* = 0.26, *P* = 0.049 and *r* = 0.45, *P* = 5 × 10^−4^, respectively; *Figure 1F*).

### Interferon regulatory factor 5 is associated with CD11c expression

**Figure 2:**
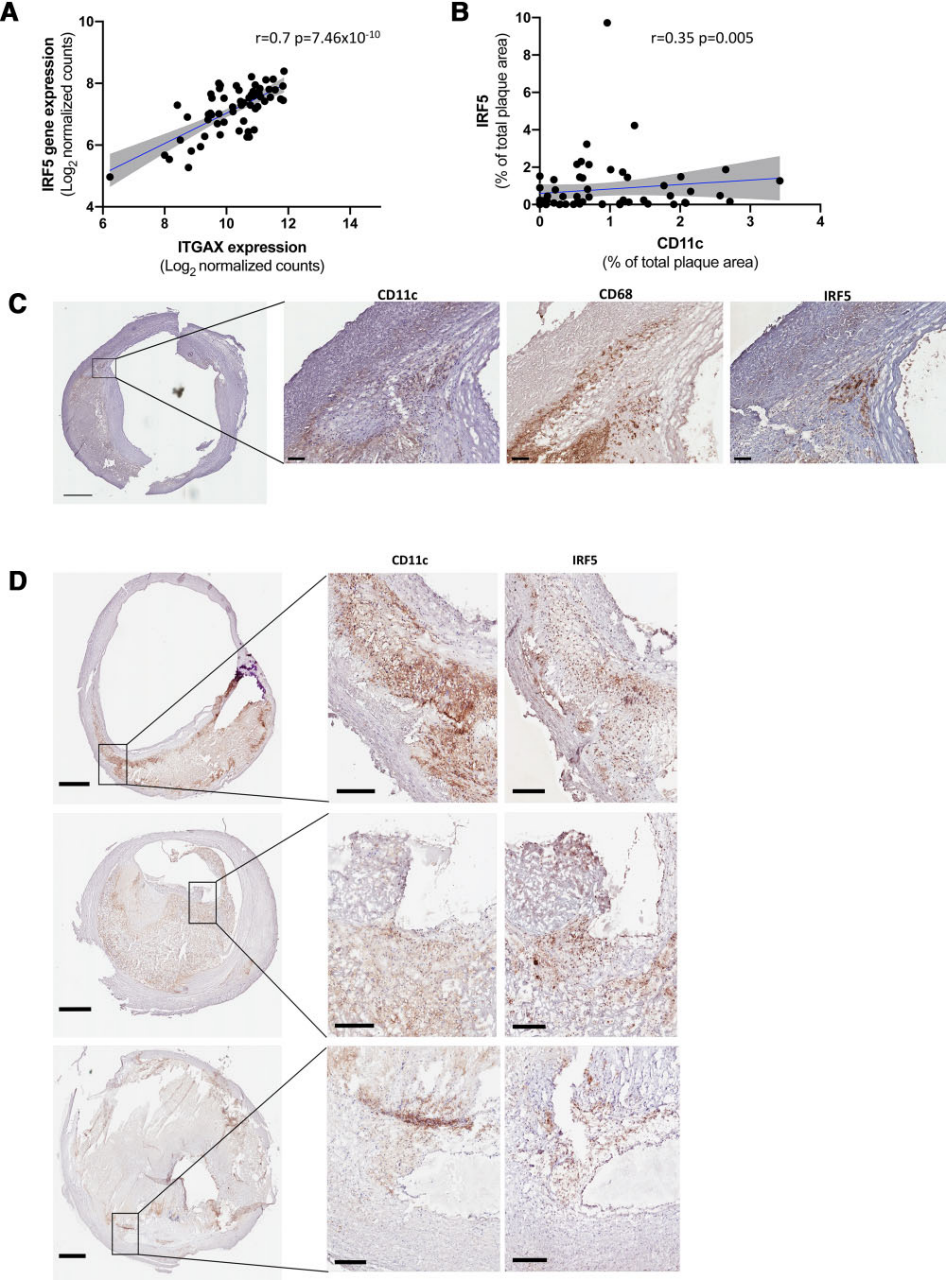
CD11c associated with IRF5 expression in human carotid plaques. (*A*) *ITGAX* (CD11c) and *IRF5* gene expression correlate strongly in human carotid plaques. Gene expression is presented as log_2_ transformed normalized counts. Spearman rank correlation test was used, *n* = 60. (*B*) Plaque area of CD11c and IRF5 (% of total plaque area) also correlated in human carotid plaques. Sections from the most stenotic region of the plaque were stained. Spearman rank correlation test was used, *n* = 62. (*C*, *D*) CD11c positive cells were identified in the same plaque regions (commonly close to the core and in the rupture prone shoulder regions) as IRF5 positive cells. CD11c (cytosol and cell membranes) and IRF5 (nuclear) positive cells are seen in brown/black. CD11c, cluster of differentiation 11c; IRF5, interferon regulatory factor 5. Scale bars 1 mm in overview images (left panels) and 200 μm in magnified areas (middle and right panels).

To explore if CD11c expression was associated with IRF5 in human carotid atherosclerosis we sought to identify whether IRF5 gene expression and plaque area correlated with CD11c gene expression. Importantly, plaque gene expression of IRF5 correlated strongly with CD11c gene expression levels (*r* = 0.7, *P* = 7.46 × 10^−10^; *Figure 2A*). Fittingly, IRF5 immunopositive plaque area correlated to CD11c immunopositive plaque area (*r* = 0.35, *P* = 0.005; *Figure 2B*) and IRF5 protein expression co-localized with CD11c protein expression in the plaque (*Figure 2C and D*; isotype controls presented in Supplementary material online, *Figure S4*). Importantly, co-localization of IRF5 and CD11c were identified in close proximity to the core region as well as in the shoulder region. The most evident co-localization was identified in the shoulder regions of the plaques (*Figure 2D*).

### CD11c is predominantly expressed by macrophages in human carotid atherosclerosis

**Figure 3:**
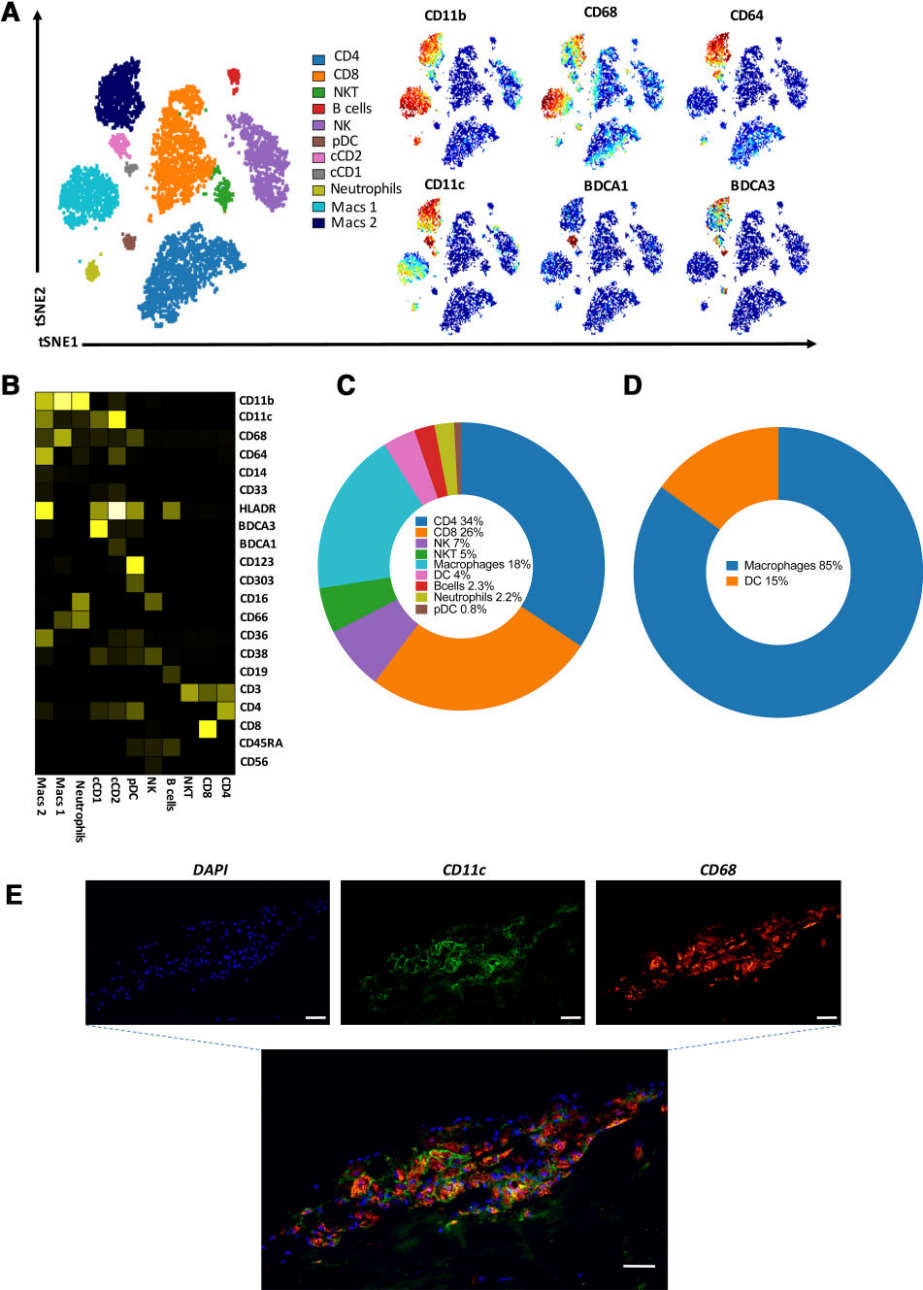
The majority of CD11c^+^ cells in human carotid plaques are macrophages. Representative CYTOF analysis of a carotid plaque with (*A*) tSNE and viSNE plots of macrophage and dendritic cell markers expression in human atheroma cells. (*B*) Heatmap visualizing the expression of markers on different CD45^+^ cell types. (*C*) Distribution of main immune cell population in human carotid plaques and (*D*) distribution of CD11c^+^ cells between macrophages and dendritic cells in human carotid plaques (*n* = 4). (*E*) Immunofluorescent double staining showing that the majority of CD11c^+^ cells in the human atherosclerotic plaque are CD68^+^. CD11c, green. DAPI, blue. CD68, red. Scale bars 50 μm. CD68, cluster of differentiation 68; CD11c, cluster of differentiation 11c; DAPI, 4′,6-diamidino-2-phenylindole.

Next, the immune landscape in human atherosclerotic plaques was characterized using mass cytometry to elucidate which cell types predominantly express CD11c. Data were analysed using an unbiased dimensionality reduction algorithm, t-distributed stochastic neighbor embedding (tSNE) on live CD45^+^ cells and single cells were clustered according to shared marker expression (*n* = 4). These analyses revealed 11 distinct clusters (*Figure 3A*) including macrophage and dendritic cell (DC) populations. Specific cell clusters were identified based on the expression of the different markers per cluster as shown in the heatmap (*Figure 3B*). T cells; CD4 and CD8T cells constitute 50% of plaque CD45^+^ cells. Myeloid cells (macrophages and DCs) come second comprising 22% of all CD45^+^ cells (*Figure 3C* and Supplementary material online, *Figure S5*). CD11c is usually considered a marker for DCs; however, it is also expressed by macrophages.^15^ Indeed, along with DCs, macrophages in human atherosclerotic plaques express high levels of CD11c as shown in the overlay plots (*Figure 3A*) and in the heatmap (*Figure 3B*). In fact, most of the CD11c-expressing cells in the human atherosclerotic plaques were identifiable as macrophages (85% vs. 15%, *Figure 3D*). Immunofluorescence triple staining on human plaque tissue confirmed that the majority of CD11c^+^ cells were also CD68 positive (*Figure 3E*).

### Interferon regulatory factor 5 regulates macrophage phenotype and function contributing to plaque inflammation and increased necrotic core size in human atherosclerosis

Interferon regulatory factor 5 and ITGAX show strong associations with gene levels of macrophage markers in our plaque RNA sequencing analysis including CD68, CSF1R, FCGR1A, and ITGAX (*r* = 0.37–0.85 and adjusted *q*-value < 0.01 (according to the two-stage linear step-up procedure of Benjamini, Krieger, and Yekutieli; Supplementary material online, *Figure S6*). No correlation was found with DC markers. Amongst T cell markers, CD4 expression, shared by human T cells and macrophages, also correlated with IRF5 and ITGAX expression (*r* = 0.67, adjusted *q* < 0.01 and *r* = 0.85, adjusted *q* < 0.01, respectively). Due lack of association with other T cell markers, the association with CD4 was likely driven by the presence of CD4^+^ plaque macrophages.^16^ Both IRF5 and ITGAX expression correlated negatively to smooth muscle cell lineage markers, ACTA2 and MYH11 [*r* = –0.51 to –0.70 and adjusted *q*-value < 0.01 (according to the two-stage linear step-up procedure of Benjamini, Krieger, and Yekutieli) for all].In line with these findings, no clear overlap of IRF5 and α-actin positive areas were detected (Supplementary material online, *Figure S7*).

**Figure 4.**
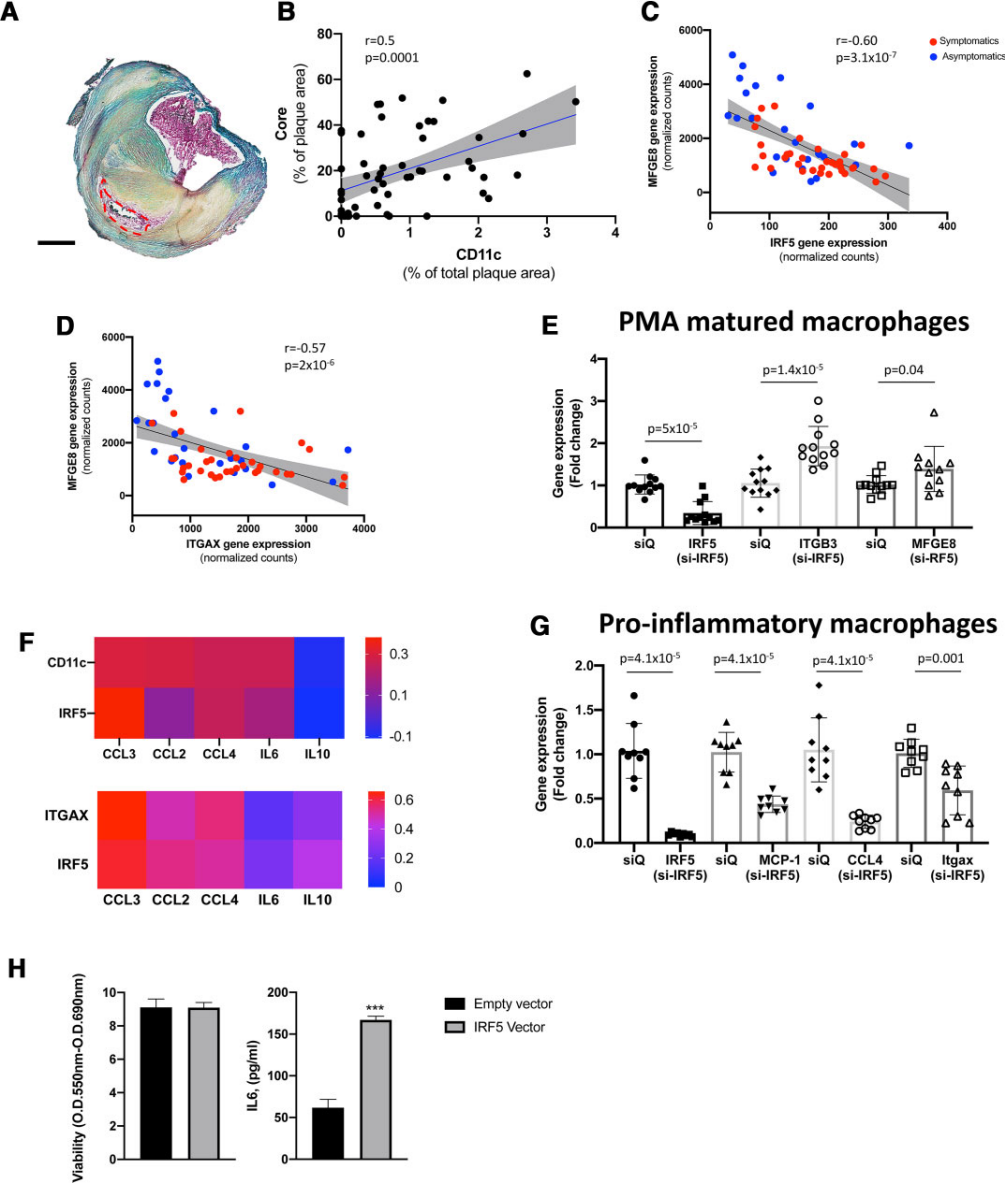
CD11c is associated with necrotic core area and reduced gene expression levels of Milk Fat Globule—Epidermal growth factor—factor VIII (*MFGE8*). (*A*) Movat pentachrome was used to identify necrotic core areas (acellular non-fibrotic tissue). Core area is marked by red-dotted line. Scale bar 1 mm (*n* = 57). (*B*) Human plaque necrotic core area correlate strongly with CD11c plaque area (*n* = 57). Data are presented as % of total plaque area. Spearman rank correlation test was used. (*C*, *D*) *MFGE8* gene expression levels correlate inversely to plaque gene expression levels of *IRF5* and *ITGAX* (CD11c). Data are presented as normalized counts. Spearman’s rank correlation coefficient was used (*n* = 60). Red indicates symptomatic patients and blue indicates asymptomatic patients. (*E*) Gene expression levels of *MFGE8* and *ITGB3* were up-regulated in THP-1 PMA matured macrophages upon IRF5 silencing, *n* = 11–12. Mann–Whitney *U* tests were used to identify significant differences. Boxes represent median levels and bars represent interquartile range. (*F*) Heatmap showing correlation coefficients for CD11c and IRF5 positive plaque area, plaque tissue gene expression of I*TGAX* and *IRF5* to plaque protein levels and plaque gene expression levels of interleukin-6 (IL6), Chemokine (C–C motif) ligand 3 (also known as macrophage inflammatory protein-1α), Chemokine ligand 4 (CCL4/macrophage inflammatory protein-1β), monocyte chemoattractant protein-1 (MCP-1/CCL2), and interleukin-10 (IL10). CD11c and IRF5 plaque area were presented as % of total plaque area. IL6, CCL3, CCL4, and MCP-1 were presented as arbitrary units/gram wet weight plaque. Interleukin-10 was presented as picogram/gram wet weight plaque. Spearman’s rho was used for correlations analyses. (*G*) Gene expression levels of *CCL2* (*MCP-1*), *CCL-4*, *ITGAX* (CD11c), and *IRF5* were reduced by *IRF5* silencing in M1 matured THP-1 cells, *n* = 9. Mann–Whitney *U* tests were used to identify significant differences. Boxes represent median levels and bars represent interquartile range. (*H*) IRF5 overexpression by adenovirus transfection of human plaque cells caused increasing release of IL6 into cell culture supernatants. Four replicates per condition were studied. Unpaired *t*-test was used to identify significant differences. siQ, silencing control.

Next, we sought to explore whether the expression of IRF5 and CD11c was associated with plaque inflammation and size of the necrotic core in human atherosclerosis. First, we assessed necrotic core (acellular and non-fibrotic areas) size in the human carotid plaques using Movat pentachrome (*Figure 4A*). A clear positive correlation between CD11c protein expression and necrotic core size was also identified in the human plaques (*r* = 0.5, *P* = 0.0001; *Figure 4B*). Interferon regulatory factor-5 induced CD11c^+^ macrophages have been shown to reduce the efferocytosis capacity by down-regulating the Mfge8-avß3 integrin pathway in mice.^10^ In line with the previous murine study, we identified strong inverse correlations of *IRF5* and *ITGAX* gene expression with *MFGE8* gene expression (*r* = –0.6, *P* = 3.1 × 10^−7^ and *r* = –0.57, *P* = 2 × 10^−6^, respectively; *Figure 4C and D*). No associations with *ITGB3* was identified, possibly due to the ITGB3 expression not being restricted to macrophages. To test if IRF5 activation affects *MFGE8* expression, THP-1 cells were matured into macrophages using PMA *in vitro*. *IRF5* was then silenced using siRNA. Interestingly, expression of both *ITGFB3* and *MFGE8* were significantly increased upon *IRF5* silencing (*Figure 4E*).

Next, to explore the association of IRF5 and CD11c with plaque inflammation gene expression levels and plaque tissue homogenate protein levels of IL-6, CCL-3, CCL-4, IL-10, and CCL-2 were assessed. Plaque protein levels of CCL-3, CCL-4, and CCL-2 were all found to correlate with CD11c^+^ and plaque area whereas IRF5^+^ plaque area only correlated significantly with CCL-3 protein levels (*Figure 4F*). Plaque gene expression levels of *IRF5* and *ITGAX* correlated with *CCL-3*, *CCL-4*, and *CCL-2* gene expression [significant gene correlations had calculated *q*-values ≤ 0.01 (FDR 1%), according to the two-stage step-up method of Benjamini, Krieger, and Yekutieli; correlations coefficients are shown in *Figure 4F*]. THP-1 derived macrophages were polarized into a pro-inflammatory phenotype and transfected with *IRF5* siRNA or left untreated. In line with the correlations identified in the human plaque tissue, cells treated with *IRF5* siRNA had significantly lower gene expression levels of *ITGAX*, *CCL-4*, and *CCL-2* (*Figure 4G*). Finally, using adenovirus transfection in isolated human atheroma plaque cell cultured we assessed if IRF5 overexpression affects the inflammatory activity. In line with our siRNA experiments, the overexpression of IRF5 human plaque cells significantly increased the release of IL-6 into plaque cell culture supernatants (*Figure 4H*).

### Interferon regulatory factor 5 deficiency reduces the frequency of plaque ruptures in mice

**Figure 5:**
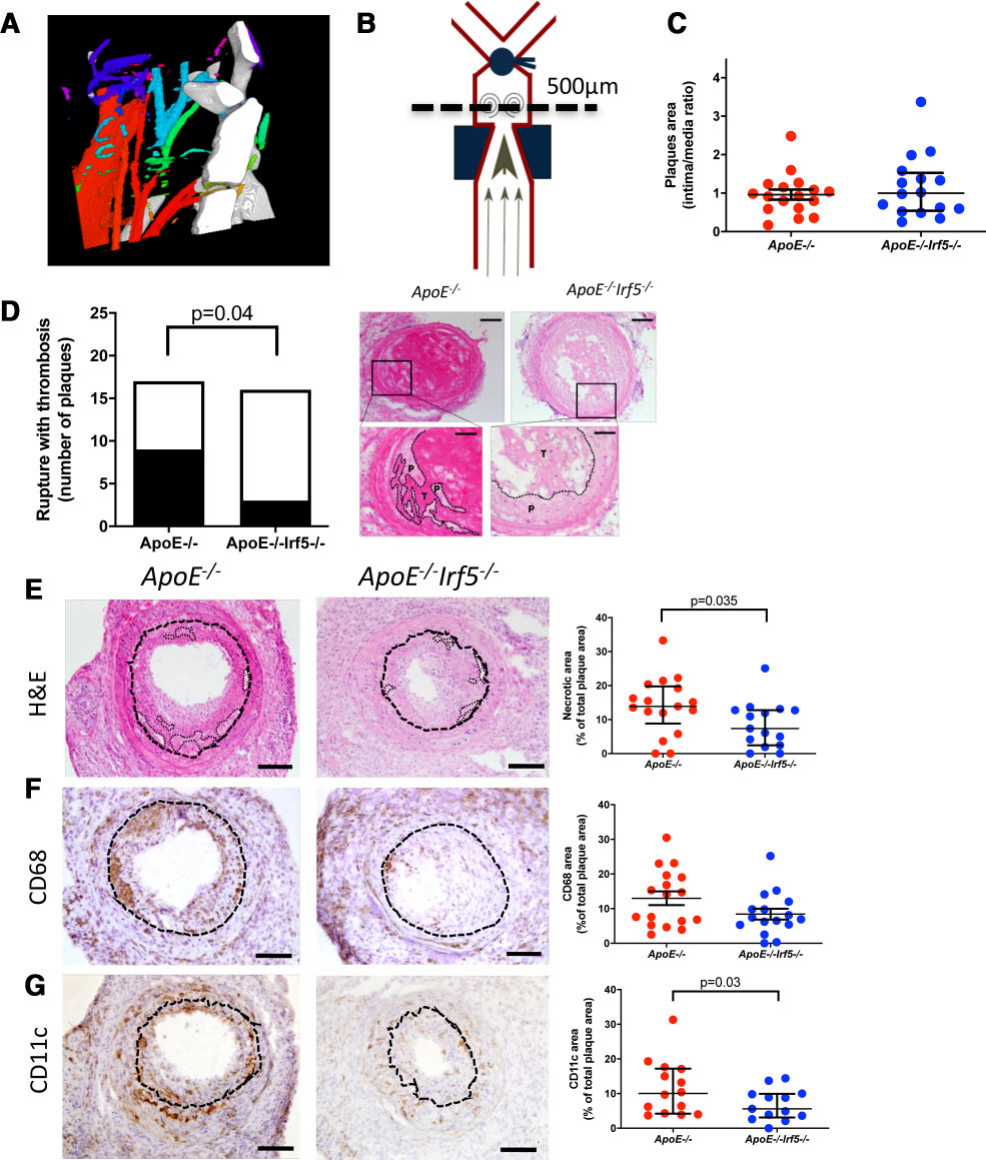
Interferon regulatory factor-5 deficiency reduced the frequency of plaque rupture and reduced both necrotic core and CDC11c plaque area in an inducible plaque rupture model. (*A*) Schematic figure of the common location of plaques ruptures identified in the area between the cast and ligation. (*B*) Computed tomography (CT) with contrast agent demonstrated that the blood flow was diminished, but not interrupted, after partial ligation proximal to the bifurcation of the carotid artery. Red indicates a high blood flow and blue and green indicate a lower blood flow. (*C*) No difference in total plaque area, intima/media ratio based on haematoxylin and eosin, was seen when comparing *ApoE* ^−/−^ and *ApoE* ^−/−^ *Irf5* ^−/−^ carotid lesions. (*D*) Plaque rupture frequencies are reduced in *ApoE* ^−/−^ *Irf5* ^−/−^ mice compared with *ApoE* ^−/−^ mice. Representative images of haematoxylin and eosin stained cross-sections of the right carotid artery 4 days after cast placement in the intra-cast region of (left) and *ApoE* ^−/−^ *Irf5* ^−/−^ mice (right). Scale bars, 100 μm. The left insert demonstrates a rupture, characterized by the loss of continuity of the fibrous cap with invasion of thrombus into the plaque tissue and at the right insert a non-ruptured plaque in *ApoE* ^−/−^ *Irf5* ^−/−^. P indicates plaque tissue and T indicates thrombus. Scale bars insert, 50 μm. Scale bar, 100 μm. (*E*–*G*) Representative images of cross-sections of carotid arteries after the cast with respective staining and quantification. (*E*) The sections were stained with haematoxylin and eosin for plaque and necrotic core size (necrotic cores are indicated with a dotted line). A decrease in necrotic core size was observed in the interferon regulatory factor-5-deficient group. (*F*) CD68 was used to identify macrophages and (*G*) CD11c. Unless mentioned otherwise, all quantifications are shown as a percentage of the cross-sectional plaque size. All values are expressed as median and interquartile range. Scale bar, 100 μm; *n* = 16–17.

As IRF5 and CD11c were strongly associated with plaque rupture in human atherosclerosis, we aimed to explore whether IRF5 deficiency would reduce plaque rupture frequency. To do so, a surgical model combining carotid artery ligation with a shear stress modifying cast was used (Supplementary material online, *Figure S1*). Upon the ligation and collar placement we confirmed continuous blood flow using computed tomography with arterial contrast agent as seen in *Figure 5A*.

To identify plaque ruptures (a disruption of the endothelium with infiltrating blood and a luminal thrombus), which commonly occurred between the cast and the ligation (*Figure 5B*), all common carotid arteries were sectioned and every 50 μm stained with haematoxylin and eosin (H&E). No significant difference in plaque size was identified comparing *ApoE* ^−/−^ and *ApoE* ^−/−^  *Irf5* ^−/−^ (*Figure 5C*). Plaque ruptures were identified in 12 out of 33 (36%). When comparing the frequency of plaque ruptures in *ApoE* ^−/−^ and *ApoE* ^−/−^  *Irf5* ^−/−^ mice, the frequency of plaques ruptures was significantly reduced in the IRF5-deficient mice (3 of 16 vs. 9 of 17, *P* = 0.04; *Figure 5D*).

### Interferon regulatory factor 5 deficiency reduces CD11c positive plaque area and the number of CD11c^+^ macrophages

Next, we assessed plaque area of necrotic core (H&E), CD68, CD11c, CD206, collagen (Picrosirius Red), and TUNEL to identify plaque phenotype changes associated with IRF5 deficiency. Importantly, necrotic core and CD11c plaque area were reduced in the IRF5^−/−^ group [6.7 (IQR 2.1–12.3)% vs. 14.7 (IQR 10.7–20.6)% of plaque area, *P* = 0.035 and 5.6 (IQR 3.1–9.9)% vs. 10.05 (IQR 4.2–17.2)% plaque area *P* = 0.03, respectively; *Figure 5E–G* and Supplementary material online, *Figure S8*].

To confirm if the reduction in CD11c was due to a loss of CD11c^+^ macrophages as we previously showed, leukocytes from the anatomically closely related deep cervical lymph nodes were isolated. Using flow cytometry (gating strategy presented in Supplementary material online, *Figure S9A*), we identified a 61% reduction in macrophages (CD11b^+^ and F4/80^+^; *P* = 0.006, Supplementary material online, *Figure S9B*) and a 72% reduction in CD11c^+^ macrophages (CD11b^+^, F4/80^+^ and CD11c^+^; *P* = 0.007, Supplementary material online, *Figure S9C*). No difference in CD11c^+^ DCs was seen (identified as CD11c^high^ and MHC-II^high^; Supplementary material online, *Figure S9D*).

## Discussion

The view that inflammation is a key component of atherosclerotic plaque formation, vulnerability, and rupture has gathered momentum in recent years.^2,3,17,18^ Macrophages are key initiators of athero-inflammation but are also crucial to the resolution stages of the inflammatory process, with their contribution depending on their state of activation.^5^ Rewiring macrophage transcriptional states is an attractive therapeutic strategy in CVD to prevent excess inflammation. We and others recently showed the importance of the transcription factor IRF5 in murine atherogenesis.^10,11^ In the present study, we demonstrate by two complementary strands of evidence in mouse and human a direct mechanistic link between the pro-inflammatory transcription factor IRF5, macrophage activation, and atherosclerotic plaque vulnerability to rupture (*Structured Graphical Abstract*).

By mining the gene expression of macrophage associated markers and transcription factors in the plaque bulk RNA sequencing in the CPIP cohort, we identify IRF5 as the transcription factor with the greatest impact on differentiating symptomatic from asymptomatic carotid disease. No significant increase of IRF5 plaque immunopositive area was found in symptomatic plaques, possibly due to divergences of kinetics between RNA and protein. Considering that asymptomatic plaques can be at high risk for rupture and that the phenotype of symptomatic plaques may change due to repair processes activated upon the rupture, we used a histological vulnerability index as a marker of rupture–prone plaques. We and others have previously demonstrated the utility of this carotid vulnerability index as a composite histological score combining markers of plaque stability and vulnerability to assess an overall risk score of the plaque phenotype and predict future cardiovascular events.^14,19–23^ This simultaneous combination of markers has previously been used by us and others and aims to provide a global balance of plaque stability, instead of relying on single markers. Interferon regulatory factor-5 expression at the protein and gene level was significantly associated with the plaque composite vulnerability index, indicating the existence of a close correlation between unfavourable plaque composition and IRF5 expression.

In earlier studies, IRF5 was shown to have a pro-atherogenic role in mouse model of lipid-driven atherogenesis,^10,11^ and in the formation of plaques with a thin-cap fibroatheroma in a shear stress modulated lesion model.^10^ These models differ from human disease, as the atherosclerotic plaques they form, rarely rupture within the timeframe of the studies. In this manuscript, we utilized an inducible plaque rupture model where a plaque disruption occurs in 50% of the mice undergoing surgery.^24–26^ This surgical model leads to the formation of vulnerable and ruptured plaques with increased necrotic core size, thinned and cracked fibrous caps, and formation of a thrombus inside the lumen (Supplementary material online, *Figure S10*). Interferon regulatory factor-5 deficiency protected from the development of plaque rupture in this model, demonstrating that IRF5 not only drives the formation of morphologically vulnerable plaques,^10^ but also induces actual plaque rupture. The effect of IRF5 deficiency in preventing carotid rupture of atherosclerotic plaques in mice and the association of IRF5 expression with unfavourable vulnerability carotid plaque index in humans demonstrate that IRF5 is a candidate therapeutic target for plaque vulnerability in humans.

Traditionally, the expression of CD11c in atherosclerosis has only been studied in the context of DCs.^27,28^ However, CD11c is a marker shared between macrophages and DCs. Using mass cytometry in murine atherosclerosis, we have recently shown that in murine aorta, CD11c is predominantly expressed by macrophages and DCs.^28^ We have also shown that aortic CD11c^+^ macrophages are the only subset of macrophages whose representation increases during atherogenesis.^29^ Aortic intima resident macrophages express CD11c and have a pro-atherogenic role in murine models.^30^ To date, less is known regarding the cellular identity of CD11c myeloid cells in the human atherosclerotic plaque. Using cytometry by time of flight, we show that CD11c^+^ macrophages are the most common CD11c myeloid cells in the human plaque. This is in agreement with our histological findings showing co-localization between CD68 and CD11c in the human atherosclerotic plaque.

We have previously shown that overexpression of IRF5 in human primary macrophages leads to a pro-inflammatory ‘M1’ macrophage polarization with up-regulation of inflammatory genes.^8,9^ We also have demonstrated that IRF5 leads to pro-inflammatory ‘M1’ macrophage polarization in murine macrophages *in vitro*, and in models of atherosclerosis and arthritis.^10,31^ We showed that in mouse IRF5 drives the transcription of the Itgax gene that encodes for CD11c, and genetic deficiency of IRF5 resulted in a loss of CD11c^+^ intralesional macrophages.^10^ In the current study, our computational analysis provides several lines of evidence that CD11c and IRF5 expression are a distinguishing feature of plaques with a high vulnerability index and/or symptomatic carotid plaques. Interferon regulatory factor-5 and CD11c are not only correlated with each other at the gene and protein level, but their expression was also positively correlated with the levels of pro-inflammatory markers. Inverse correlations between *IRF5* and *ITGAX* with *MFGE8* gene expression were identified, in agreement with our previous study where IRF5-deficient mouse macrophages had an increased efferocytic capacity through up-regulation of *Mfge8* and *Itgb3* (both important factors in efficient efferocytosis).^10^ These positive and negative associations were reproducible *in vitro* (*Figure 4*). Finally, IRF5 gene expression was significantly positively correlated with the expression levels of macrophage marker genes, but not DCs. In absence of correlations with other lymphocyte markers, correlation between the expression of IRF5 and CD4 could be due to the expression of CD4 by human plaque macrophages.^16^ Interestingly, strong negative correlations were observed between IRF5, ITGAX, and smooth muscle cell markers, in keeping with immunohistochemistry data (Supplementary material online, *Figure S7*) reinforcing the link between IRF5 and human vulnerable plaque biology. Our RNASeq and functional data indicate that the relevance of IRF5 to human vulnerable plaque biology is linked to its ability to regulate macrophage phenotype and activation.

Consistently, in an inducible model of carotid artery plaque rupture, a reduction in necrotic core size and CD11c expression in the lesion was observed in *ApoE* ^−/−^  *Irf5* ^−/−^ mice when compared with *ApoE* ^−/−^ mice, without a significant effect on total CD68 or CD206 expression indicating a specific loss of CD11c expression on plaque macrophages. This loss in the plaque was mirrored by a loss of CD11c macrophages, but not DCs, in the draining deep cervical lymph nodes. The evidence that CD11c expression, macrophage content, and necrotic core plaque area are affected by IRF5 deficiency in the murine model of inducible plaque rupture supports the notion that the lower number of plaque ruptures is due to prevention of IRF5 induced changes in plaque composition and macrophage phenotype. Taken together, our data demonstrate that CD11c^+^ macrophages are an IRF5-driven pro-inflammatory subset of macrophages that are enriched in plaques vulnerable to rupture.

The shoulder region is known to be an active area of the plaque located at the boundary between plaque and normal artery, and a common location for a rupture to occur.^32^ According to Richardson *et al*.^32^ nearly two-thirds of all plaque ruptures were identified in the shoulder region which was also confirmed in an intravascular ultrasound study by Maehara *et al*.^33^ IRF5 staining was largely contained within CD11c immunopositive carotid plaque areas generally surrounding the core of the plaque with a predilection for the shoulder regions of the plaques. In concert with the evidence that IRF5 deficiency prevents plaque rupture in mice, our findings implicate IRF5 in enhanced inflammation in the shoulder region, leading to plaque rupture.

There is today no clear evidence from genome-wide association studies (GWAS) supporting the role of IRF5 in CVD. Genome-wide association studies had shown that IRF5 downstream variants were strongly associated with coronary artery disease (leading variant rs11556924, *n* = 1 543 070, *P* = 5.5 × 10^−38^; https://t2d.hugeamp.org). However, this variant was about 1 Mb far away from gene body of IRF5. Whether IRF5 played a definite causal role need further investigation, e.g. Mendelian randomization. An upstream variant rs9972727 of CD11c showed strong association with CVD (*P* = 1.0 × 10^−12^).^34^ Interestingly, this variant was also an eQTL SNP which regulated gene expression of CD11c (*P* = 9.4 × 10^−207^) in human blood which suggested that CD11c may be implicated in CVD.^35^ Yet, the lack of strong GWAS evidence does not rule out that IRF5 induced CD11c^+^ macrophages are important mediators in the formation of rupture–prone atherosclerotic plaques.

Our study is not without limitations. There is not a single experimental model of atherogenesis that reproduces all facets of human CVD. The inducible plaque rupture model has been well described and used to induce plaque ruptures in mice, despite its imperfections to mimic human disease.^24–26^ This model could not allow the study of plaque erosion which is responsible for ∼25–40% of cardiovascular complications.^36–38^ A second limitation of the study is that we could not identify association between risk factors such as diabetes and IRF5 or CD11c expression in the carotid plaques due to the relatively limited number of patients included. Such association was previously demonstrated in peripheral blood cells.^39^ The lack of associations in our study could likely be explained by the fact that the diabetes patients included in the present study had a rather good glycaemic control (median HbA1c levels 56 mmol/mol). Discrepancies in the plaque areas positive for CD11c and IRF5 immunostaining can likely be explained by the fact that IRF5 expression is inducible and dependent on the state of activation and polarization of macrophages.^7,8^ Furthermore, the glucose levels in the plaque tissue may not necessarily reflect circulating glucose levels, as several factors might affect the local glucose concentration (e.g. plaque size), factors affecting the diffusion of glucose (e.g. endothelial permeability and neovessel density), and parameters modulating glucose metabolism (e.g. numbers of cells in the plaque and state of activation).^14^

## Conclusions

In conclusion, we show that IRF5 expression is linked to symptoms and features of plaque vulnerability in human carotid endarterectomies in the CPIP biobank. Using a murine model of inducible carotid artery plaque rupture, we show that IRF5 drives not only the formation of morphologically vulnerable plaque, but also their actual rupture. We also demonstrate that the role of IRF5 in murine and human plaque instability is inextricably linked to the generation of IRF5^+^ CD11c^+^ pro-inflammatory macrophages. Our study progresses our knowledge on IRF5 in atherosclerosis towards translation by offering validation of IRF5 as a potential therapeutic target in human CVD.

## Supplementary material

Supplementary material is available at *European Heart Journal* online.

## Supplementary Material

ehab920_Supplementary_Data
