# Supplementary material for: Interferon regulatory factor-5-dependent CD11c+ macrophages contribute to the formation of rupture–prone atherosclerotic plaques
Source: Eur Heart J. 2022 Feb 9;43(19):1864–77. doi: 10.1093/eurheartj/ehab920 (PMC9113304; doi:10.1093/eurheartj/ehab920)
Supplement: ehab920_Supplementary_Data [file ehab920_supplementary_data.pdf]

SUPPLEMENTARY MATERIAL

for

"IRF5 induced CD11c^{+} macrophages contribute to rupture prone atherosclerotic plaques"

Supplementary methods and materials

Human plaque RNA sequencing

RNA was prepared using standard Trizol method and cleared of Ribosomal RNA using Ribo-Zero™ Magnetic Kit (Epicentre). Next, the strand specific paired end RNAseq libraries were prepared with ScriptSeq™ v2 RNA-Seq Library v2 Preparation Kit (Epicentre) and paired end libraries were sequenced using high-output kit version 2, HiSeq2000 platform, Illumina, USA. Bases with Phred score less than 20 and adapter sequences or bases were removed using TrimGalore (Version 0.3.7, Babraham Bioinformatics, Cambridge, UK). Further, quality controls were performed using FastQC (Version 0.11.2, Babraham Bioinformatics, Cambridge, UK) and summarized using mutliqc (Version 0.9). Of 60 sequenced samples, 47 samples were histologically scored for vulnerability index and thus selected for multivariate analyses. Gene expression was quantified using Salmon with gene annotations from Gencode v.27 and imported using tximport. Using count data, differential expression analysis was examined using DESeq2 and counts were normalized using DESeq2´s median of ratios and log2 transformed.

For orthogonal partial least squares discriminant analysis (OPLS-DA), gene expression of macrophages associated genes and transcription factors were pulled out from whole dataset and analysed in SIMCA-P software package (version 14.1, Umetrics, Umeå, Sweden) using symptomatic status as response variable. Prior to analysis, the gene expressions were mean-centered and scaled to unit variance. R^2Y and Q^2Y metrics described the percentage of variation explained by the model and predictive ability of the model, respectively. Furthermore, overall contribution of each gene to group discrimination was ranked by VIP values (variable influence on projection). Gene with VIP value greater than 1 was considered important in group discrimination.

For network analysis, counts were normalized between samples using a trimmed mean of M-values (TMM) by the edgeR, resulting in gene expressions as log2-counts per million.(1)

Network analysis of 60 human plaque expression data was conducted using the weighted gene coexpression network analysis (WGCNA). Genes with low-expression variance (<1.5) across samples were filtered out, resulting in 2092 genes.(2) A power of 5 was chose which is the lowest power to fulfil the scale-free network ($R2 \geq 0.9$). Gene co-expression network was then constructed, and clusters of genes (modules) were identified consequently. Pearson correlation between the first principal component of gene expressions in each module and symptomatic status were calculated. Pathway enrichment in each module was performed using Enrichr on 1721 high-quality pathways from the Elsevier Pathway Collection. (3)

Histology, immunohistochemistry and

Stained sections submerged in 60% isopropanol and stained for neutral lipids (Oil Red O) in 0,4% Oil Red O in 60% isopropanol. Macrophages (CD68), smooth muscle cells (α-actin) and intra plaque haemorrhage (glycophorin A/CD235) were stained using a primary monoclonal mouse anti-human CD68 antibody (KP1, 1:100 in 10% rabbit serum), a primary mouse anti-human α-actin antibody (1A4, 1:50 in 10% rabbit serum) and a primary monoclonal mouse anti-human glycophorin A/CD235a antibody (1:400 in 10% rabbit serum; all from DakoCytomation, Glostrup, Denmark). A biotinylated rabbit anti-mouse F(ab')2 antibody fragment (1:200 in 10% rabbit serum; DakoCytomation, Glostrup, Denmark) was used as the secondary antibody. Plaque collagen levels were assessed by Russell-Movat Pentachrome staining technique using alcian blue, hematoxylin, crocein scarlet-acid fuchsin and 6% alcoholic saffron. Irf5 and CD11c were stained using mouse anti human IRF5 (Abcam, 20μg/mL) and mouse anti human CD11c (Abcam, 1μg/mL). Following incubation with an appropriate secondary antibody or MACH3 mouse polymerase kit (Biocare Medical), 3,3'-diaminobenzidine (DAB) was used for staining detection and nuclei were counterstained with Mayer ́s hematoxylin. Positively stained plaque areas (% of total plaque area) were scanned and

photographed with Aperio image scope v.8.0 (Aperio, Vista Californien, USA), then blindly quantified using Biopix iQ 2.1.8 (Gothenburg, Sweden).

Immunofluorescence staining was performed on 8µm OCT embedded carotid sections which were fixed in acetone and blocked for 45 min on RT in PBS containing 1%BSA, 10% goat serum and 0.3M glycine. After blocking sections were incubated with a cocktail of mouse anti human-CD11c (5µg/ml; Abcam, ab11029) and rabbit antihuman-CD68 (0.365µg/ml; Cell signalling technology, clone D4B9C, #76437S) antibodies overnight at 4oC, followed by mix of donkey anti-rabbit alexa 555 (ab150106, Abcam, Cambridge, UK) and goat anti-mouse alexa 488 (ab150077, Abcam, Cambridge, UK) antibodies for 45 min on RT (both at 1 µg/ml). Thereafter, sections were treated with 0.03% Sudan black B (ICN Biomedicals) and mounted with vectashield mounting medium with DAPI (Vector Laboratories). Specificity of each antibody was confirmed by staining with respective IgG isotype control antibodies (rabbit IgG1, abcam ab172730; and mouse IgG1, ab18443) at similar concentrations. Finally, slides were viewed and imaged by Nikon Eclipse E800 fluorescent microscope equipped with Olympus DP74 camera.

Luminex and Proximity extension assay analyses of human carotid plaques

Luminex immunoassay (Millipore Corporation, MA) and the Olink Proseek Multiplex CVD96x96 kit (Clinical Biomarkers Facility, Science for Life Laboratory, Uppsala University) were used for plaque cytokine assessment, as previously described.(4) The Luminex analysis was performed according to the manufacturer´s instructions as previously described.(5)

In-vitro IRF5 silencing

IRF silencing was performed in human monocytic THP-1 cells, grown in RPMI-1640 media supplemented with 10% heat-inactivated fetal bovine serum, 1% pen-strep, and 0.05 mM β-

mercaptoethanol at 37°C under 5% $CO2$. Silencing of IRF5 was performed on PMA differentiated THP-1 cells using siRNA. Briefly, $2x10^5$ THP-1 cells were grown in 12 well plate in 1 ml culture media and incubated with 50 ng/mL of PMA at 37 °C for 24 h to transform into macrophages. Following this cell were transfected overnight either with 30 nM scrambled siRNA (Allstars negative control siRNA; 1027281; Qiagen) or siRNAs targeting human IRF5 (HSS142676; stealth siRNA IRF5; ThermoFisher Scientific) and cultured subsequently in complete media for 48 h. Next, IRF5 silenced macrophages were polarized to proinflammatory phenotype by exposing them with 20 ng/ml of interferon gamma (IFNG) for 24 h in RPMI conditioned media with 10% FBS. Subsequently cell were lysed for mRNA extraction and evaluated for gene expression levels of levels of *IRF5* (Hs00158114_m1); *ITGB3* (Hs01001469_m1); *MFGE8* (Hs00170712_m1); *MCP-1* (Hs00234140_m1); *CCL4* (Assay ID); *ITGAX* (Hs00174217_m1) and *ACTIN* (Hs99999903_m1) by real time PCR (Viia 7 real time PCR system, Applied biosystem).

Human atheroma cell culture transfection

Single cell suspension was obtained through enzymatic digestion of human carotid plaque tissue using a previously published protocol.(6) Briefly, the tissue was washed in RPMI, finely minced and incubated in collagenase type I (400 units/ml), elastase type III (5 units/ml), and DNase (300 units/ml), with 1 mg/ml soybean trypsin inhibitor, 2.5 μg/ml polymixin B, and 2 mM $CaCl2$, in a shaker at 37°C for 45 min. Cells were filtered through a 80-μm Nylon mesh, washed, counted and resuspended in RPMI 5%FBS. Cells were seeded at a density of 10^6 cells/ml in 96 well plate and left to settle for 2 hrs. At that time, media was replaced with serum and antibiotic -free media and cells were infected with either a first-generation replication-deficient adenovirus serotype 5 containing human IRF5 (AdIRF5) or a control mock vector (AdC) at a multiplicity of infection (moi) of 200:1 for 2 hrs after which FBS supplemented

media was added to the wells.(7) Four replicates per condition were studied. Cells were cultured for an additional 48 hrs after which supernatants were removed for IL6 measurement (duplicates) using commercially available ELISA and cellular viability was assessed by using 3-(4,5- dimethyl-2-yl)-2,5-diphenyltetrazolium (MTT; Sigma) assay following manufacturer instructions.

Surgical plaque vulnerability model

As mice do not develop vulnerable plaques per se a surgical model combining carotid artery ligation and placement of a shear stress modifying cast was used as described. (8,9) In summary, the right common carotid artery was ligated proximal to the bifurcation at 9-weeks of age. Four weeks later a shear stress modifying cast (Promolding) was placed proximal to the ligation. The gradually constrictive cast (inner diameter decreasing from 500 to 250 μm) induces low shear stress (LSS) proximal to the cast, high shear stress (HSS) in the intra-cast region and oscillatory shear stress (OSS) distal to the cast (Supplementary Figure 1). Four days after the cast was placed all mice were sacrificed by a barbiturate overdose. Carotid arteries were embedded in Optimum-Cutting Temperature compound (CellPath) and immediately snap-frozen in liquid nitrogen.

X-ray computed tomography of mice carotid arteries

To confirm continuous blood flow upon the ligation and the cast placement contrast-enhanced computed tomography (CT) was performed. Viscover ExiTron nano 12000 (Miltenyl Biotech) contrast agent was injected intra venous (100 μL) 5 minutes prior to scanning and the scanning. Scanning (resolution 10 um/pixel) was performed using a quantum FX micro-computed tomography (microCT; PerkinElmer). Mice were anesthetized with isoflurane prior to scanning and images were processed and blood flow was color coded using ImageJ 1.51d (NIH).

Mouse tissue preparation and morphological analysis of carotid arteries

The entire length of the carotid artery was sectioned into 5 μm cryosections. Sections from every 250 μm was stained with haematoxylin and eosin to assess necrotic core size (defined by acellular and nonfibrotic areas >300 μm2), plaque area (intima-media ratio) and plaque rupture. Picrosirius Red (Polysciences) according to manufacturer's instructions was performed to detect plaque collagen. Images were obtained using an Olympus BX51 osteometric brightfield & fluorescence microscope (Olympus). Lesion size was quantified using ProgRes CapturePro 2.5 (Jenoptik) and collagen as well as core area were quantified by Clemex Vision Lite v5.0 (Clemex). Plaque ruptures were defined as disruption of the cap, blood infiltrating the plaque in combination with overlying luminal thrombus. Plaque ruptures were confirmed by two operators independently. All analyses were performed by blinded operators.

Immunohistochemistry of mouse carotid arteries

For the immunohistochemical analysis rat anti-mouse CD206 (clone MR5D3, Bio-Rad). hamster anti-moue CD11c (clone N418, Bio-Rad) and rat anti-mouse CD68 (clone FA-11, 1:200, AbD Serotec) antibodies were used. Biotinylated goat anti-rabbit (1:400), goat anti-hamster (1:400) and rabbit anti-rat (1:400) were used as a secondary antibody (all Vector Laboratories) and slides were counterstained with Harris Hematoxylin (Surgipath). Apoptotic cells were visualized by fluorescein-dUTP using the TdT-mediated dUTP-X nick end labeling (TUNEL) according to manufacturer's instructions (Roche). Anti-mouse α-actin-Cy3 (clone 1A4, Sigma-Aldrich) counterstained with DAPI was used to stain vascular smooth muscle cells. Images were obtained as described above and immunopositive areas were quantified using Clemex Vision Lite v5.0 (Clemex).

Flow cytometry

Single cell suspensions of the deep cervical lymph nodes were obtained using a 70 µm cell strainer for flow cytometric analysis and the following antibodies were used: CD11b-BV711 (clone M1/70, 1:200), CD11c-APC (clone N418, 1:100), CD3-FITC (clone 145-2C11, 1:100), CD4-PE-cy7 (clone RM4-5, 1:200), CD45-APC-cy7 (clone 30-F11, 1:200), F4/80-BV605 (clone BM8, 1:50, all Biolegend) and MHC-II-BV421 (clone M5/114.15.2, 1:250, BD Pharmingen). Fluorescence minus one (FMO) controls were used for all gates. Flow cytometric analysis was performed on a BD LSR II flow cytometer (BD Biosciences). Data analysis were performed using FlowJo software 10.07 (FlowJo).

Supplementary references

1. Robinson MD, McCarthy DJ, Smyth GK. edgeR: a Bioconductor package for differential expression analysis of digital gene expression data. Bioinformatics. 2010;26:139-40.
2. Langfelder P, Horvath S. WGCNA: an R package for weighted correlation network analysis. BMC Bioinformatics. 2008;9:559.
3. Chen EY, Tan CM, Kou Y, Duan Q, Wang Z, Meirelles GV, Clark NR, Ma'ayan A. Enrichr: interactive and collaborative HTML5 gene list enrichment analysis tool. BMC Bioinformatics. 2013;14:128.
4. Rattik S, Wigren M, Björkbacka H, Fredrikson GN, Hedblad B, Siegbahn A, Bengtsson E, Schiopu A, Edsfeldt A, Dunér P, Grufman H, Gonçalves I, Nilsson J. High plasma levels of heparin-binding epidermal growth factor are associated with a more stable plaque phenotype and reduced incidence of coronary events. Arterioscler Thromb Vasc Biol. 2015;35:222-8.
5. Edsfeldt A, Gonçalves I, Grufman H, Nitulescu M, Dunér P, Bengtsson E, Mollet IG, Persson A, Nilsson M, Orho-Melander M, Melander O, Björkbacka H, Nilsson J. Impaired fibrous repair: a possible contributor to atherosclerotic plaque vulnerability in patients with type II diabetes. Arterioscler Thromb Vasc Biol. 2014;34:2143-50.
6. Monaco C, Andreakos E, Kiriakidis S, Mauri C, Bicknell C, Foxwell B, Cheshire N, Paleolog E, Feldmann M. Canonical pathway of nuclear factor kappa B activation selectively regulates proinflammatory and prothrombotic responses in human atherosclerosis. Proc Natl Acad Sci U S A. 2004;101:5634-9.
7. Krausgruber T, Saliba D, Ryzhakov G, Lanfrancotti A, Blazek K, Udalova IA. IRF5 is required for late-phase TNF secretion by human dendritic cells. Blood. 2010;115:4421-30.

8. Sasaki T, Kuzuya M, Nakamura K, Cheng XW, Shibata T, Sato K, Iguchi A. A Simple Method of Plaque Rupture Induction in Apolipoprotein E-Deficient Mice. Arterioscler Thromb Vasc Biol. 2006;26:1304-1309
9. Magné J, Gustafsson P, Jin H, Maegdefessel L, Hultenby K, Wernerson A, Eriksson P, Franco-Cereceda A, Kovanen PT, Gonçalves I, Ehrenborg E. ATG16L1 Expression in Carotid Atherosclerotic Plaques Is Associated With Plaque Vulnerability. Arterioscler Thromb Vasc Biol. 2015;35:1226-35.

Supplementary table 1. List of antibody clones used in CyTOF analysis.

150Nd	CD1c/BDCA1	L161	BioLegend	In-house
167Er	CD197/CCR7	G043H7	Fluidigm	Fluidigm
144Nd	CD11b	ICRF44	Fluidigm	Fluidigm
162Dy	CD11c	Bu15	Fluidigm	Fluidigm
151Eu	CD123/IL-3R	6H6	Fluidigm	Fluidigm
160Gd	CD14	M5E2	Fluidigm	Fluidigm
173Yb	CD141/BDCA3	1A4	Fluidigm	Fluidigm
164Dy	CD15/SSEA-1	W6D3	Fluidigm	Fluidigm
148Nd	CD16	3G8	Fluidigm	Fluidigm
165Ho	CD163	GH1/61	Fluidigm	Fluidigm
142Nd	CD19	HIB19	Fluidigm	Fluidigm
154Sm	CD3	UCHT1	Fluidigm	Fluidigm
147Sm	CD303/BDCA2	201A	Fluidigm	Fluidigm
169Tm	CD33	WM53	Fluidigm	Fluidigm
152Sm	CD36	5-271	Fluidigm	Fluidigm
167Er	CD38	HIT2	Fluidigm	Fluidigm
145Nd	CD4	RPA-T4	Fluidigm	Fluidigm
89Yb	CD45	HI30	Fluidigm	Fluidigm
153Eu	CD45RA	HI100	Fluidigm	Fluidigm
172Yb	CD57	HCD57	Fluidigm	Fluidigm
146Nd	CD64	10.1	Fluidigm	Fluidigm
149Sm	CD66a	CD66a-B1.1	Fluidigm	Fluidigm
171Yb	CD68	Y1/82A	Fluidigm	Fluidigm
168Er	CD8a	SK1	Fluidigm	Fluidigm
161Dy	CD90/Thy-1	5E10	Fluidigm	Fluidigm
174Yb	HLA-DR	L243	Fluidigm	Fluidigm
176Yb	CD56/NCAM	NCAM16.2	Fluidigm	Fluidigm

Supplementary figure 1

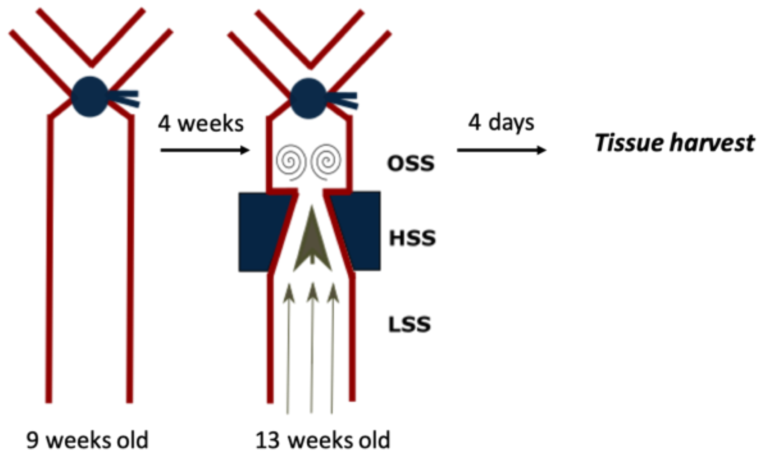

Supplementary figure 1. Description of the surgical model. At 9-weeks of age the right carotid was ligated and after 4 weeks a cast was placed proximal to the ligation to induce shear stress changes for four days before sacrifice and tissue harvest. Low shear stress (LSS) proximal, high shear stress (HSS) in the intra-cast region and oscillatory shear stress distal to the cast.

Supplementary figure 2

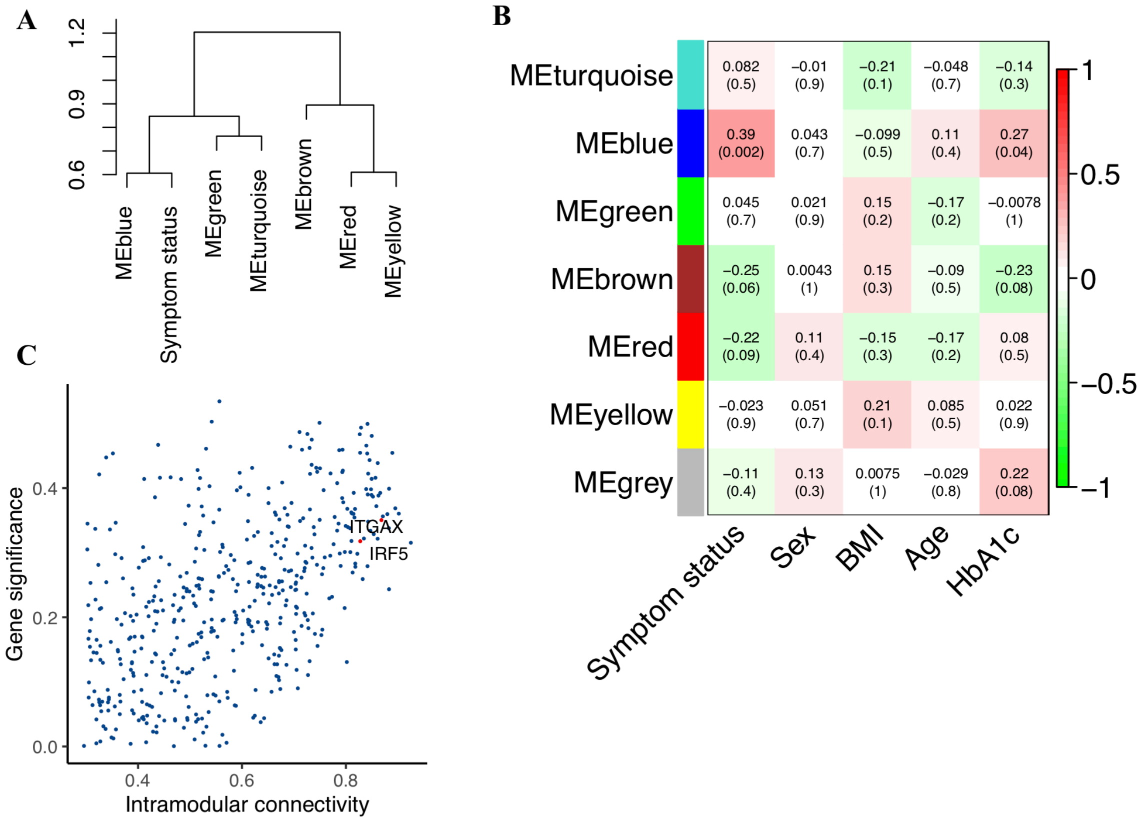

Supplementary Figure 2. IRF5 and ITGAX were co-expressed in a module that is associated with symptomatic status A) A hierarchical clustering dendrogram of the module eigengenes**.** Y-axis represents dendrogram height which is a distance metric. **B)** Relationships of module eigengenes and clinical traits. Each row in the table corresponds to a module, and each column to a trait. Numbers in the table report the correlations of the corresponding module eigengenes and traits, with the p-values printed below the correlations in parentheses. The table is color coded by correlation according to the color legend. Genes that didn't fall into any modules were assigned to the grey module. 1-symptomatic and 0-asymptomatic. 0-male and 1-female. **C)** Gene significance in the association with symptomatic status and high intramodular connectivity in blue module. IRF5 and ITGAX are red color annotated.

Supplementary figure 3

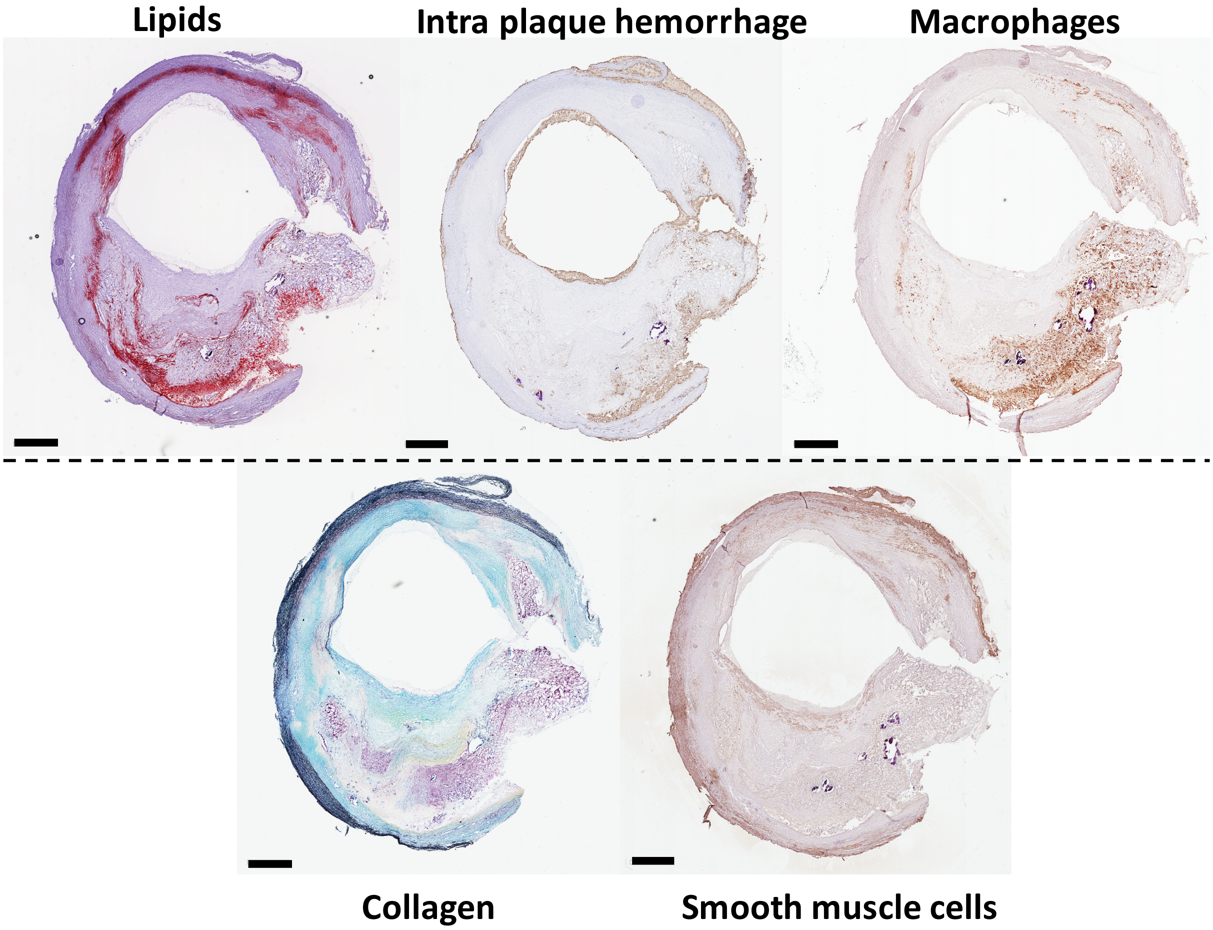

Supplementary figure 3. Description of the calculated vulnerability index. Vulnerability index was calculated by dividing the sum of the plaque area stained positive of CD68, Oil red O and glycophorin A with the sum of the plaque area stained positive for alpha-actin and collagen.

Supplementary figure 4

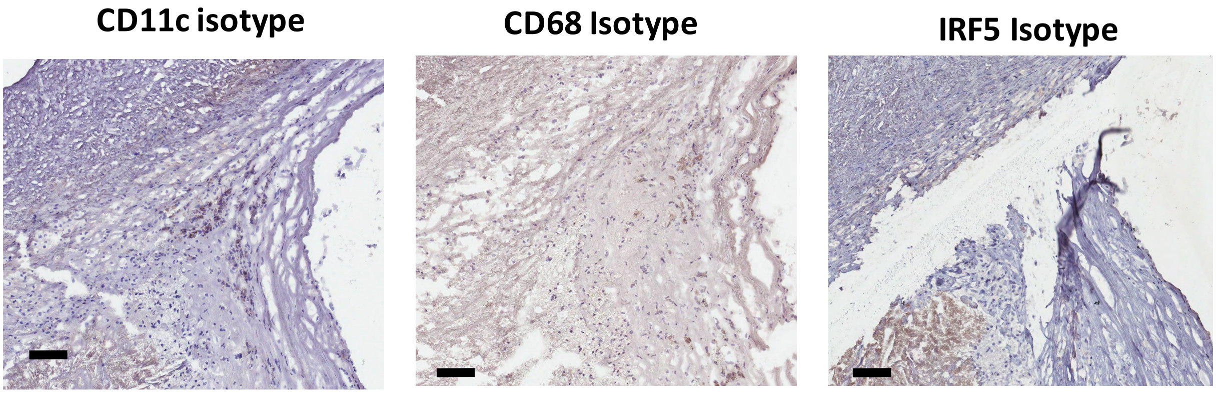

Supplementary figure 4. Isotype controls. Images showing isotype controls for CD11c, CD68 and IRF5. Scalebars 100 um.

Supplementary figure 5

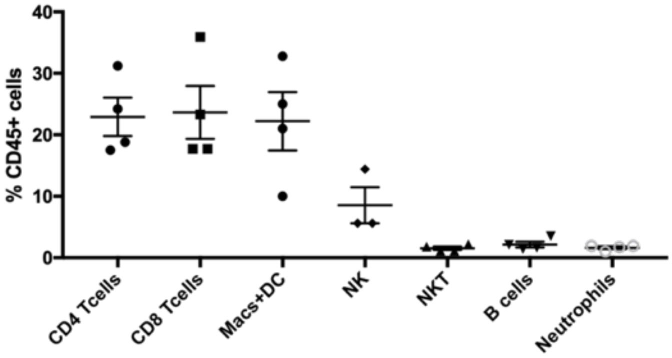

Supplementary figure 5. Distribution of all CD45+ cell types as assessed in CYTOF in 2 human plaques. CYTOF mass cytometry of human carotid plaques revealed that T-cells are dominant phenotype of all CD45+ cells present in the human atherosclerotic plaques. n=4. Data presented as mean and SD.

Supplementary figure 6

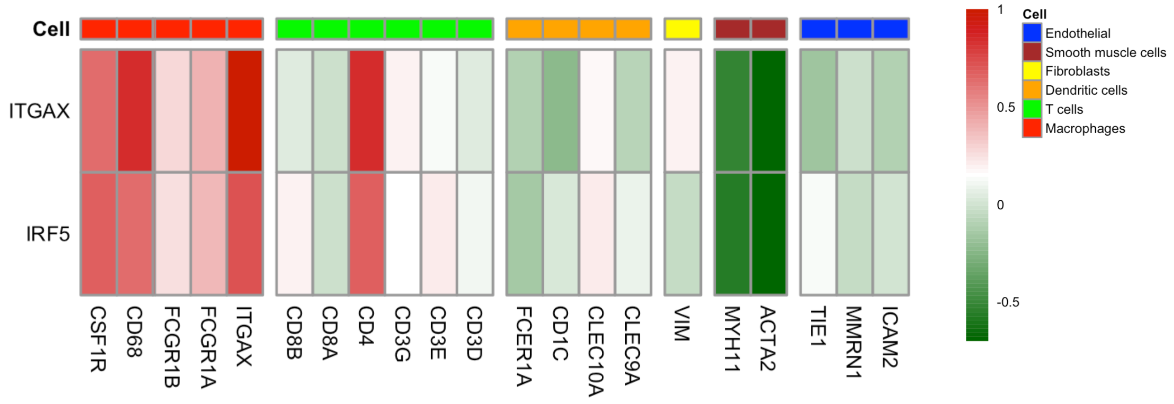

Supplementary figure 6. Heatmap displaying correlation coefficients between ITGAX, IRF5 and cell markers commonly used to identify endothelial cells (ICAM2, MMRN1, TIE1), smooth muscle cells (ACTA2, MYH11), fibroblasts (VIM), T-cells (CD3C, CD3E, CD3G, CD4, CD8A, CD8B), dendritic cells (CLEC9A, CLEC10A, CDC1C, FCER1A) and macrophages (ITGAX, FCGR1A, FCGR1B, CSF1R and CD68) as assessed by human plaque bulk RNA-sequencing. Spearman correlation coefficient was used. n=60.

Supplementary figure 7

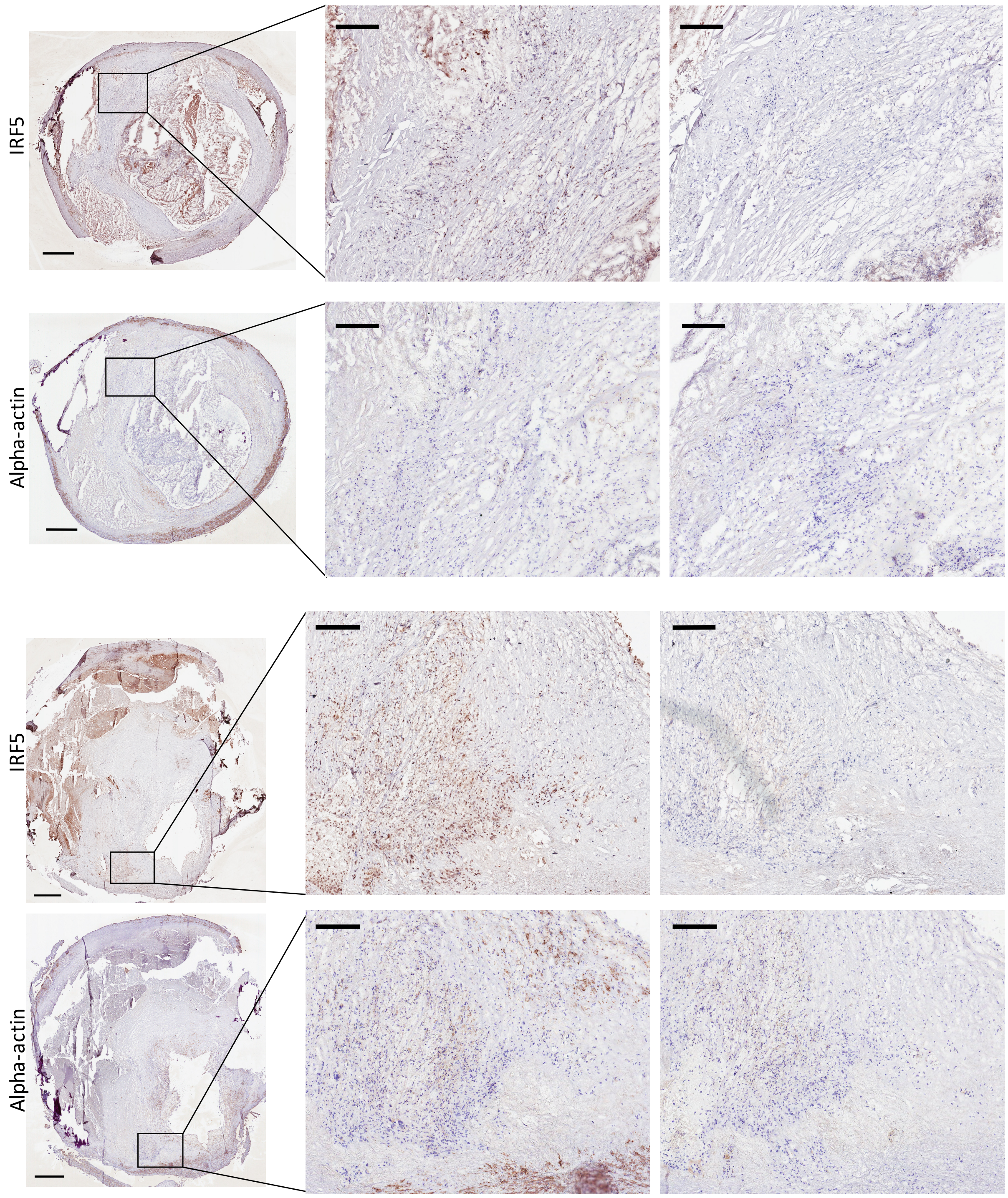

Supplementary figure 7. Immunohistochemistry for IRF5 and alpha-actin on human carotid plaques did not show a clear overlap of IRF5+ and alpha-actin+ plaque areas. IRF5 and alpha-actin positive cells in brown. Right panel showing proper isotype controls. Scale bars 1mm (left panel) and 200um (middle and right panels).

Supplementary figure 8

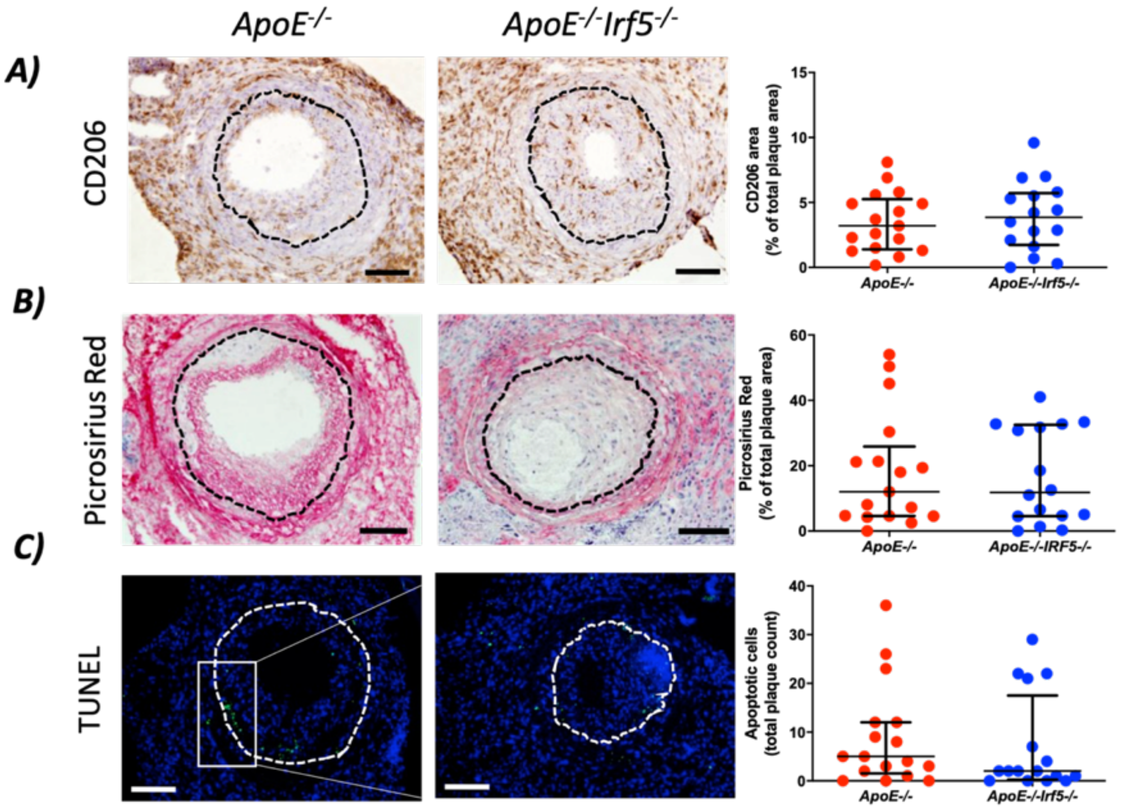

Supplementary figure 8. Irf5 deficiency did not affect CD206, collagen or plaque cell apoptosis. No difference in **A)** M2 macrophage marker CD206 area, **B)** collagen area (Picrosirius red positive in red), **C)** in total number of apoptotic cells (TUNEL) were identified when comparing ApoE-/- and ApoE-/- Irf5-/- carotid lesions. Representative images from the right carotid artery 4 days after cast placement in the intra-cast region. ApoE-/- lesions to the right and and ApoE-/- Irf5-/- lesions to the left.

Scale bar,100 µm. Unless mentioned otherwise, all quantifications are shown as a percentage of the cross-sectional plaque size. All values are expressed as median and IQR.

Supplementary figure 9

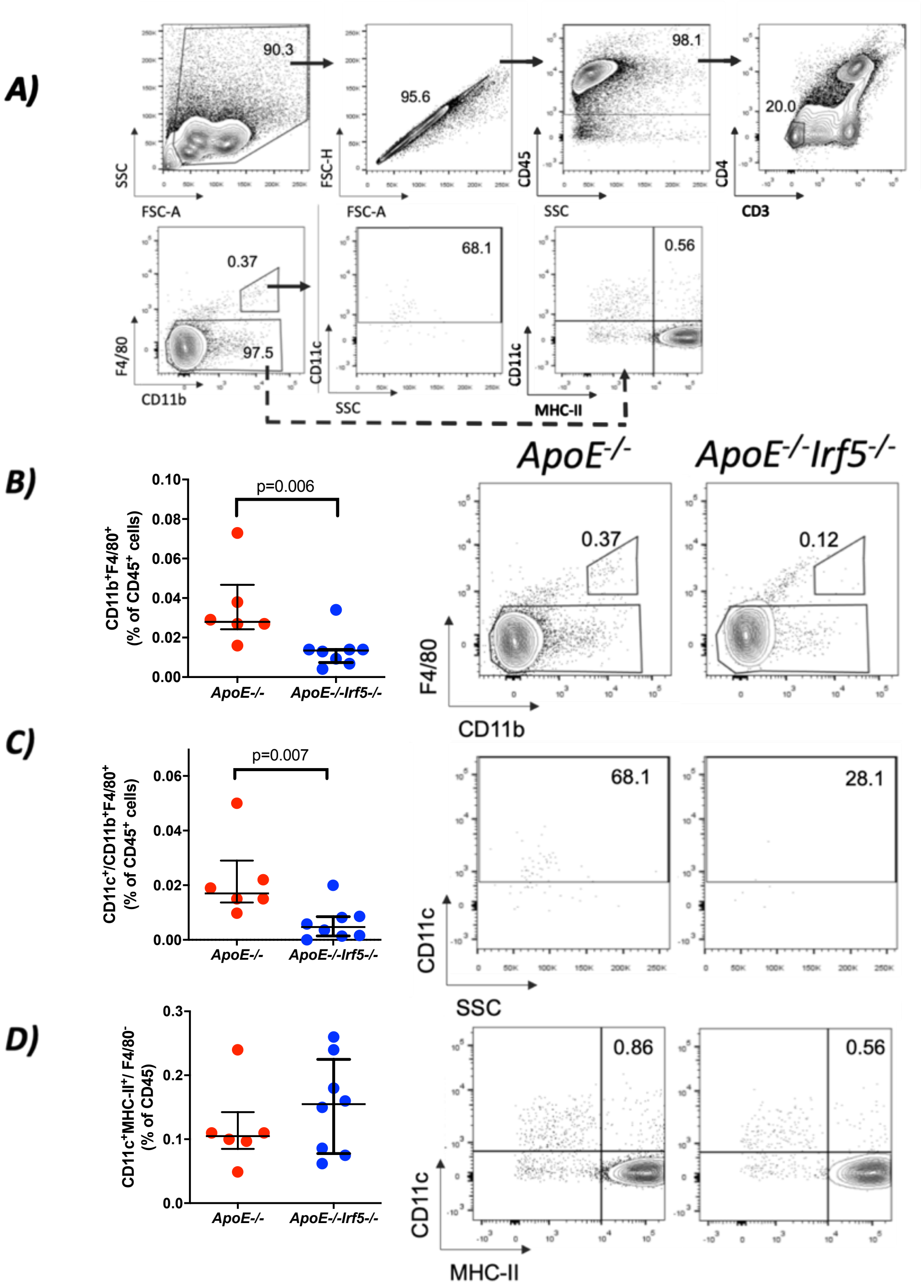

Supplementary figure 9. ApoE-/-Irf5-/- mice have a reduction in CD11c macrophages in the deep cervical lymph nodes. A) The gating strategy for identification of macrophages, CD11c+ macrophages and dendritic cells. The percentage of **B)** macrophages (CD45+, CD11b+ and F4/80+) and **C)** CD11c+-macrophages (CD45+, CD11c+, CD11b+ and F4/80+) were reduced in ApoE-/-Irf5-/- mice compared to control mice (ApoE-/-). No significant

difference in % of **D)** dendritic cells (CD45+, CD11c+, MHC-II+, F4/80-) were identified. Representative dot plots are shown. All values are expressed as mean±SEM. **, P<0.01. Data presented as percentage (%) of CD45+ cells. N=6 in the ApoE-/- group and n=8 in the ApoE-/-Irf5-/- group.

Supplementary figure 10

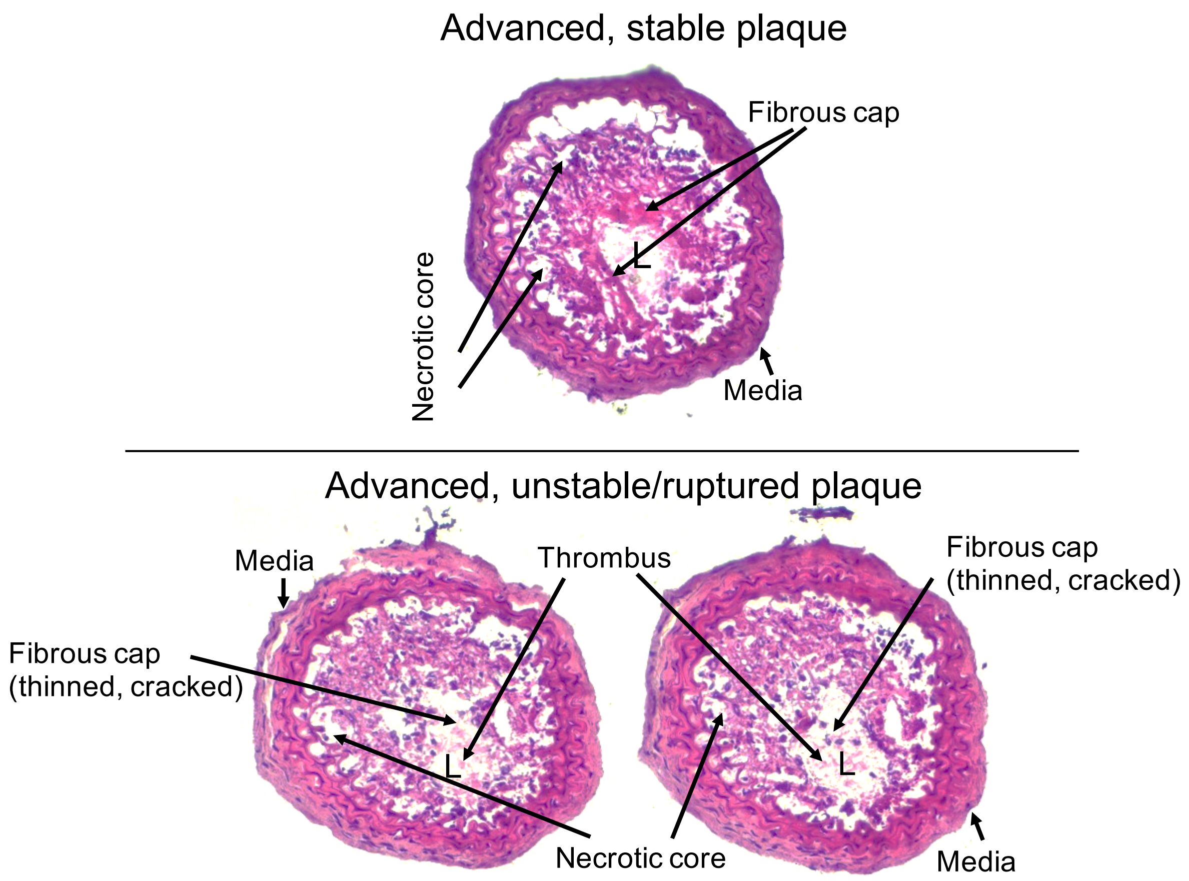

Supplementary figure 10. Characteristic features of the inducible plaque rupture model in ApoE$^{-/-}$ mice displayed using H&E staining. Upper panel shows a advanced but still stable plaque with an increased necrotic core and thick fibrous cap and an approx. 80% stenosis. Two images on the bottom indicate advanced and unstable/ruptured plaques with increased necrotic core size, thinned and cracked fibrous caps and formation of a thrombus inside the lumen (L).
